# Synthetic community derived from grafted watermelon rhizosphere provides protection for ungrafted watermelon against *Fusarium oxysporum* via microbial synergistic effects

**DOI:** 10.1186/s40168-024-01814-z

**Published:** 2024-06-05

**Authors:** Yizhu Qiao, Zhendong Wang, Hong Sun, Hanyue Guo, Yang Song, He Zhang, Yang Ruan, Qicheng Xu, Qiwei Huang, Qirong Shen, Ning Ling

**Affiliations:** 1 Key Lab of Organic-Based Fertilizers of China and Jiangsu Provincial Key Lab for Solid Organic Waste Utilization, Nanjing Agricultural University, Nanjing, 210095 China; 2 Centre for Grassland Microbiome, State Key Laboratory of Herbage Improvement and Grassland Agro-Ecosystems, College of Pastoral Agriculture Science and Technology, Lanzhou University, Lanzhou, 730020 China; 3 Plant-Microbe Interactions, Department of Biology, Science4Life, Utrecht University, Padualaan 8, Utrecht, 3584 CH the Netherlands

**Keywords:** Synthetic community, Community simplification, Disease suppression, Interspecific synergy

## Abstract

**Background:**

Plant microbiota contributes to plant growth and health, including enhancing plant resistance to various diseases. Despite remarkable progress in understanding diseases resistance in plants, the precise role of rhizosphere microbiota in enhancing watermelon resistance against soil-borne diseases remains unclear. Here, we constructed a synthetic community (SynCom) of 16 core bacterial strains obtained from the rhizosphere of grafted watermelon plants. We further simplified SynCom and investigated the role of bacteria with synergistic interactions in promoting plant growth through a simple synthetic community.

**Results:**

Our results demonstrated that the SynCom significantly enhanced the growth and disease resistance of ungrafted watermelon grown in non-sterile soil. Furthermore, analysis of the amplicon and metagenome data revealed the pivotal role of *Pseudomonas* in enhancing plant health, as evidenced by a significant increase in the relative abundance and biofilm-forming pathways of *Pseudomonas* post-SynCom inoculation. Based on in vitro co-culture experiments and bacterial metabolomic analysis, we selected *Pseudomonas* along with seven other members of the SynCom that exhibited synergistic effects with *Pseudomonas*. It enabled us to further refine the initially constructed SynCom into a simplified SynCom comprising the eight selected bacterial species. Notably, the plant-promoting effects of simplified SynCom were similar to those of the initial SynCom. Furthermore, the simplified SynCom protected plants through synergistic effects of bacteria.

**Conclusions:**

Our findings suggest that the SynCom proliferate in the rhizosphere and mitigate soil-borne diseases through microbial synergistic interactions, highlighting the potential of synergistic effects between microorganisms in enhancing plant health. This study provides a novel insight into using the functional SynCom as a promising solution for sustainable agriculture.

Video Abstract

**Supplementary Information:**

The online version contains supplementary material available at 10.1186/s40168-024-01814-z.

## Introduction

The rhizosphere is considered as the area of soil surrounding plant roots and also the reservoir of soil microorganisms [1]. The interactions that occur within the diverse rhizosphere microorganisms and plant roots, including probiotic and pathogenic types, significantly influence plant physiological performance, highlighting the vital role of the rhizosphere microbiome in plant health and growth [2–5]. Therefore, the targeted manipulation of crop microbiota, especially the rhizosphere microbiome, emerges as an efficacious strategy to optimize sustainable crop production in the future.

The overuse of chemical fertilizers and the intensive application of monocropping practices have disrupted the balance of soil microbial ecosystems, leading to disrupted populations of beneficial microorganisms and aggravating the prevalence of crop diseases [6, 7]. Notably, the resistant crop (e.g., grafted plants) [8] show fewer disease symptoms caused by soil-borne pathogens like *Fusarium oxysporum* compared with susceptible crop (e.g., ungrafted plants) [9]. Rhizosphere microbiota transplantation (RMT) stands out as another potent tool in plant disease management. Previous studies have demonstrated that RMT from resistant donors can positively modulate the protective microbiota in soil and help suppress wilt disease [10]. These phenomena are attributed to the enrichment of specific protective microorganisms in the rhizosphere, that might be involved in conferring resistance against pathogen invasion [11]. Recent studies have emphasized the concept of a “core microbiome” in plant rhizospheres. The core microbiome comprises persistent, highly abundant, adaptable microbial species (with characteristics like wide ecological niche, efficient cooperative interactions, and tolerance to various habitats) closely linked to the host plant across diverse environments [12–15]. Within microbial communities, some taxa, known as generalists, can adapt to several conditions and thrive in various environments or habitats, such as the gut or soil [15–17]. The generalists persist across multiple habitat-specific assemblages and are typically part of the core microbiome. However, it is important to note that several findings and inferences regarding the core microbiome are derived from association analyses of “omics” data [14, 15]. The practical application of generalists as a core microbiome for suppressing soil-borne diseases warrants further validation and investigation.

Furthermore, previous studies have primarily focused on introducing a single core strain under sterile soil conditions. However, natural environments contain complex native microbiomes. These native microbiomes can significantly influence the effectiveness and behavior of the introduced strains [18, 19]. In contrast to the traditional one-strain-at-a-time approach, synthetic communities (SynComs) have emerged as effective tools for enhancing plant health and conferring resistance in non-sterile environments [17, 20]. SynComs comprise core microorganisms that work together to achieve specific functions such as disease control, salt tolerance, and improved plant growth [17, 20, 21]. SynComs are often designed and constructed based on these microbial interactions [22]. Given the high complexity of metabolites in non-sterile environments (especially in the rhizosphere), metabolite exchanges might drive species interactions in plant microbiomes, such as the synergistic interactions arising from cross-feeding [23]. Synergistic interactions in consortia have shown promising potential in promoting plant growth and mitigating stress [24–26], suggesting their applications in designing probiotic communities. However, there is still some debate around the optimal construction of SynComs using core microbiota from healthy rhizospheres and their effectiveness in enhancing plant growth and health.

Watermelon is undeniably one of the most important horticultural crops worldwide, with an annual yield of approximately 118 million tons. However, the occurrence of fungal diseases due to continuous monoculture, such as the watermelon Fusarium wilt disease, which is caused by the watermelon-specific pathogen *Fusarium oxysporum* f. sp. niveum (*F. oxysporum*), has been extensively reported [8]. Notably, grafted watermelon, obtained through the application of the grafting technology, exhibits the ability to modify the underground microbial community structure of the plant [9]. This modification leads to resistance against watermelon Fusarium wilt even in long-term continuous cropping fields. Given that, we questioned how grafted watermelon plant-associated microbial communities respond to *F. oxysporum* infection. Furthermore, we remained unclear about the extent of the protective effects of a SynCom derived from the core microbiome of grafted watermelon rhizosphere in a non-sterile environment, especially against biotic stresses, such as a high pathogen abundance. Moreover, the development of an efficient methodology for constructing and simplifying a functional SynCom remains uncertain.

Our approach involved assessing the differences between the rhizosphere microbial community structure and *F. oxysporum* densities of ungrafted and grafted watermelon plants. Then, we conducted extensive bacterial isolations from the rhizosphere of grafted watermelon, selecting core bacteria based on their widespread distribution and adaptability across varying soil fertility gradients. Next, we assembled a SynCom comprising 16 core bacterial species and assessed its effectiveness in enhancing plant health within a non-sterile environment, particularly in the presence of biotic stresses. Metagenomic and amplicon sequencing approaches were used to delve into the mechanisms underlying the beneficial effects of the SynCom. Furthermore, we developed an efficient methodology for constructing and simplifying a functional SynCom based on the synergetic interactions among the SynCom members. This study suggests a novel approach to control plant diseases and provides valuable insights into the sustainability of watermelon cultivation and industry.

## Methods

### Field experiment design and soil sample collection

The field experiment was conducted in the Nanjing Institute of Vegetable and Flower Science (31°43′ N, 118°47′ E), Nanjing, China. The region experiences a subtropical monsoon climate with an average annual temperature of 14.7°C. The soil type in the area is classified as yellow brown soil. The area of the watermelon field was 56 m^2^, and it was divided into two regions: one with continuous cropping of ungrafted watermelon and the other with continuous cropping of grafted watermelon (gourd rootstock). Each region comprised two plots. A random block design was adopted. In the area, spring and autumn planting seasons have been practiced annually since 2014. During the planting process, 16 seedlings were transplanted into each plot. Throughout the growing season, the average daily temperature reached 38°C, while the average nighttime temperature was 24°C. The watermelon fields have been continuously cultivated for eight years (16 seasons) until 2022.

In October 2022, during the flowering stage of the watermelon plants, plant rhizosphere soil samples were obtained from both the continuous cropping of ungrafted watermelon treatment (referred to as ungrafted) and the continuous cropping of grafted watermelon treatment (referred to as grafted). In detail, 7 plants with similar growth were selected from each plot (a total of 28 plants from all four plots). Thus, 14 plants for each watermelon treatment were selected. Loose adhering soil was gently shaken off and the samples were securely transported back to the laboratory in ice. The root samples were placed in 50-mL centrifuge tubes, and a suitable volume of sterile water was added to the tubes such that the roots were submerged. The samples in tubes were shaken for 30 min at 180 rpm. Then the roots in the centrifuge tubes were removed, and the tubes were centrifuged at 4000 × *g* for 10 min. The centrifuged pellets were retained as the rhizosphere soil. Finally, seven rhizosphere soil samples were obtained for each watermelon treatment (two individual samples are mixed into one for each treatment). All the rhizosphere samples were stored at − 80°C before DNA extraction.

### DNA extraction, sequencing and real-time qPCR

Total DNA of rhizosphere soil was extracted using FastDNA™ soil DNA extraction kit (MP Biomedicals, Cleveland, OH, USA). DNA concentration was then measured using the Qubit® DNA concentration test kit. Seven rhizosphere soil samples of each watermelon treatment were selected for16S rRNA gene (V4–V5 region) sequencing (LC-Bio Technology Co., Ltd., Shanghai, China). Real-time qPCR (qPCR) was used to determine the abundances of *F. oxysporum* of rhizosphere soil according to previously described protocols with minor modifications [27]. For detailed qPCR protocols, see Supplementary Methods 1.1.

### Isolation and identification of culturable bacteria

The bacteria associated with the field grafted watermelon rhizosphere were isolated as described previously with minor modifications [28]. For detailed isolation protocol, see Supplementary Methods 1.2. Bacterial full-length 16S rDNA was subsequently sequenced at LC-Bio Technology Co., Ltd. Sequence alignment was performed on the NCBI website, and each microbial species was preserved in 30% glycerol (v/v) at − 80°C.

### Incubation of culturable bacteria in soils with fertility gradients

This study aimed to investigate the composition of the microbial community in different habitat characterized by soil with varying fertility gradients (mixed in different proportions from high and low fertility soils) to identify the core microbes. Briefly, the soil with high and low fertility was mixed in nine mixing ratios, and all the isolated bacteria were inoculated in equal volume and OD_600_ into each mixing treatment. For detailed experimental design, see Supplementary Methods 1.3. After 90 days of incubation in the dark, total DNA was extracted from all treatment soil samples using FastDNA™ soil DNA extraction kit, followed by 16S rRNA gene absolute quantitative sequencing (Genesky Biotechnologies, Inc., Shanghai, China). For detailed absolute quantitative sequencing methods, see Supplementary Methods 1.4.

### Niche width

We used the “Shannon–Wiener” index to analyze niche width. This method reflects the number of different habitats that each species occupies and the evenness with which they occur [29]. Taxa that live widely in different fertility soils habitats have higher niche width values. Therefore, taxa with higher and lower niche width values can be regarded as generalists and specialists, respectively [30]. Niche width was calculated in R using the “spaa” package.

### Constitution of the bacterial SynCom

Based on the incubation experiment in soil with fertility gradients, 16 microbes that exhibited high niche width (generalists) and widely existed in 9 soil habitats were selected as core microbes for the SynCom construction. Sanger sequencing of the full-length 16S rRNA genes provided the V4–V5 region of each isolate, which could then be mapped back to ASVs of the identified 16 core microbes. Thus, representative strains for 16 core microbes can be identified (Table S1). Then, each of the 16 core strains was inoculated in beef extract peptone broth (NB) medium and incubated overnight at 37℃ at 170 rpm. The fermentation broth of each strain was centrifuged at 4000 × *g* for 5 min and re-suspended in sterile water with OD_600_ adjusted to 0.1. The suspensions of the 16 strains were mixed in equal volume (v/v) to establish the SynCom. The pathogen *F. oxysporum* strain used in this experiment was stored in glycerol at − 80°C in our laboratory. The frozen *F. oxysporum* strain was first grown on solid potato dextrose agar (PDA) for 48 h at 28°C. Then, it was transferred to fresh potato dextrose broth (PDB) and cultured for 7 days at 28°C. The *F. oxysporum* spores were collected from the PDB quantified using a hemocytometer and diluted to 10^6^ spores mL^−1^.

### Disease control by the SynCom

The effects of SynCom on the incidence of watermelon Fusarium wilt disease were investigated using greenhouse pot experiments. Briefly, the ungrafted watermelon plants seedling (cultivar: Zaojia 8424) were inoculated with either the SynCom (SBC), *F. oxysporum* (FON), both SynCom and *F. oxysporum* (SBC + FON), or sterile water (control, CK). The pot experiment included three independent biological replicates, each containing nine technical replicates. Greenhouse pot experiments were performed using a randomized complete block design. Each replicate was run in polypropylene pots filled with 600 g of dry, non-sterile soil with no history of watermelon cultivation. The pot experiments were run in a greenhouse (daytime: 16 h long at 30°C, night: 8 h long at 26°C) located in Nanjing Agriculture University. A week after inoculation of the SynCom, the *F. oxysporum* suspension was inoculated. Six weeks after the pathogen infection, we measured disease indexes [31], the aboveground height of the plant, fresh weight, dry weight, and root weight (for detailed disease indexes measuring method, see Supplementary Methods 1.5). Ungrafted watermelon rhizosphere soil samples of pot experiments were collected as described previously. DNA extraction and qPCR were performed as described previously. Eight rhizosphere soil samples of each inoculation treatment were randomly selected for 16S rRNA gene (V4–V5 region) sequencing as described previously. Three rhizosphere soil samples of each inoculation treatment were randomly selected for metagenome sequencing (LC-Bio Technology Co., Ltd.).

### Bioinformatics analyses

For 16S rRNA gene amplicon sequencing (the primer see Supplementary Methods 1.6), the raw data were first performed to quality control. The paired-end reads were merged using the USEARCH [32]. High-quality sequences were chosen via the “fastq_filter” command, followed by dereplication. The singletons were eliminated utilizing the USEARCH-unoise3 algorithm, and any chimeric sequences were excluded using the “uchime_ref” command [32]. The remaining sequences were utilized to generate an amplicon sequencing variant (ASV) table, and taxonomic assignments were carried out using the Ribosomal Database Project (RDP) classifier. Statistical and visualization of taxon annotation results using R software (V4.3.1). Co-occurrence network analysis was performed following the Molecular Ecological Network Analyses Pipeline [33]. The network was visualized using Gephi [34].

The quality of raw metagenomic sequencing data was assessed with FastQC (V0.11.9) [35], followed by quality control using Trimmomatic (V0.39) to remove adapters and low-quality sequences. Metaspades (V3.15.0) was used for sequence assembly into contigs [36], while Prodigal (V2.6.3) was used to predict coding sequences (CDS) [37], and CD-HIT was used for de-redundancy [38]. Salmon (V1.4.0) was used to map non-redundant CDS to clean reads for quantitative calculation [39], and transcripts per million (TPM) were obtained. Non-redundant CDS protein sequences were used to annotate the genome classification database (GTDB database) to obtain community species classification information [40]. Kyoto Encyclopedia of Genes and Genomes (KEGG) database was used for functional annotation [41]. The Reporter Score method was used for pathway enrichment analysis [42]. Gene sequences were also annotated against the carbohydrate-active enzymes (CAZy) database with the HMMSEARCH software and the eggNOG databases (V5.0) via eggnog-mapper (V1.0.3). The meta-links method was used to correlate species information with functional information through open reading frames (ORFs) with the same identifiers, i.e., ORF IDs. For detailed meta-links method, see Supplementary Methods 1.7.

### In vitro interactions among the core microbes

#### Supernatant assay

To explore the interactions among the core microbes, we performed an in vitro strain-paired supernatant assay. Specifically, each of the 16 core strains was inoculated in 20 mL of NB medium and incubated at 37℃ at 170 rpm for 48 h till they reached a plateau phase. The bacterial cultures were then centrifuged at 8000 rpm for 10 min, and the supernatant was filtered through a 0.22-μm sterile filter to obtain the spent medium (SM) of each strain. The cultures of each strain were then adjusted to the same OD_600_ value (= 0.1) and inoculated into the SM (1%, v/v) of all strains. The resultant cultures were incubated at 37°C for 48 h. For control, the adjusted cultures of each strain were also inoculated separately into a fresh NB medium. At the end of the incubation, we measured OD_600_ of all cultures. The result was expressed as the OD_600_ of the culture of a strain in different SM divided by the OD_600_ of the control culture of the strain (i.e., OD_600_ spent/fresh). Three replicates for each treatment.

#### Determination of microbial interactions

Microbial interactions, whether positive, neutral, or negative, were determined by measuring OD_600_ spent/fresh [24]. Specifically, to categorize the interactions among the strains, we used the *t*-test to compare whether the OD_600_ spent/fresh of three independent experiments was significantly greater than or less than 1. The interaction between two strains was categorized as either positive ( +) or negative ( −) if OD_600_ spent/fresh was significantly greater than and lower than 1 (*P* < 0.05), respectively. For the remaining samples (*P* > 0.05), the interaction was categorized as neutral (0).

### Metabolome analysis

The strains were cultured in a fresh NB medium as described above. Late-stage stable bacterial supernatants and their extracellular metabolites were collected. Sterile fresh medium was used as the control. LC–MS/MS analyses were performed using a UHPLC system (Vanquish, Thermo Fisher Scientific) with a UPLC BEH Amide column (2.1 mm × 100 mm, 1.7 μm) coupled to Orbitrap Exploris 120 mass spectrometer (Orbitrap MS, Thermo). For detailed chromatography protocol, see Supplementary Methods 1.8.

### Effects of simplified SynComs on plant health

Eight core strains (Table S1) were mixed in equal volume and OD_600_ to construct a simplified SynCom named SSC8. In addition, the 16 core bacterial strains were ranked based on their degree (the number of edges that connect to a node) in the co-occurrence network (Fig. S13). The top four and top eight strains in terms of degree were selected, and they were mixed in equal volumes and OD_600_ to construct synthetic communities named SSC4D and SSC8D, respectively (Table S1). SSC4D and SSC8D groups contained *P. aeruginosa* Q6, and they were used as control treatments (i.e., other methods to simplify SynCom). Meanwhile, we also designed treatments to inoculate the same volume of sterile water (CK) and the initial SynCom (SBC). The strains used for each treatment are shown in Table S1. The composition of the synthetic bacterial communities, source of pot soil, planting methods, and management practices are described above. To investigate whether the simplified SynComs impacted plant growth, none of the treatments in this potting experiment were supplemented with pathogen. After 6 weeks, plant height, root weight, fresh weight, and dry weight of the aboveground portion were measured.

### Statistical analyses

The α-diversity of bacterial communities was estimated using the Shannon and Richness index. A principal coordinate analysis (PCoA) based on Bray–Curtis distance metrics was used to explore the differences in bacterial community compositions. The distinct and shared bacteria were analyzed with Venn diagrams using the “VennDiagram” package in R. Analysis of variance (ANOVA) was used to determine the statistical significance of multiple comparisons using the “agricolae” package in R. The plots were created using the “ggplot2” package in R. The figure legends contain a comprehensive description of the detailed statistical analysis.

## Results

### Field disease incidence assessment and SynCom development

Assessment of watermelon wilt disease incidence in the field revealed that the ungrafted watermelon plants exhibited notably higher wilt disease incidence than the grafted watermelon plants (Fig. S1a). Furthermore, the *F. oxysporum* density was significantly lower in the rhizosphere of the grafted plants than the ungrafted plants (Fig. S1f). The rhizosphere microbiota of ungrafted plants significantly differed from that of grafted plants (Fig. S1). To explore the disease-suppressing potential of the rhizosphere microbiota of the grafted plants and develop an effective disease-suppressing SynCom, we established a library of 394 bacterial isolates from the rhizosphere of field grafted watermelon plants (Fig. S2). Since grafted plants exhibit superior disease suppression, the bacterial isolates from the rhizosphere microbiota of the grafted plants were selected as candidates for the SynCom (Fig. S3). Our results demonstrated that there were significant differences in both soil physical and chemical properties and bacterial community composition across habitats (nine different fertility gradients soils) (Supplementary Methods 1.3; Fig. S4; Table S2). Given that, we assessed the ecological niche width of individual microbes by classifying them as either generalists (top 10%, 32 amplicon sequence variants (ASVs)) or specialists (bottom 10%) (Fig. S5a, b). Furthermore, 67 ASVs (common ASVs) were commonly found across all nine habitats (Fig. S5c). Based on our definition of core microbes as those that shared between generalists and common ASVs, we identified 16 core microbes (16 clusters of 97% similarity originating from 32 ASVs; Fig. S5d; Table S3). Notably, after 90 days of culture, these 16 microbes were still present in all nine habitats and had high relative abundance (Fig. S5e). Although these microbes contributed only 5% of the overall richness, they accounted for almost 75% of the total absolute abundance at the genus level annotation (Fig. S6). Sanger sequencing of the full length 16 S rRNA gene amplicon provided the V4–V5 region of each isolate which could then be mapped back to ASVs of core microbes. Ultimately, representative strains for 16 core microbes can be identified (Table S1; Table S3), including *Pseudoxanthomonas mexicana* Q1, *Pseudorhodoferax aquiterrae* Q2, *Rhizobium daejeonense* Q3, *Lysobacter panacisoli* Q4, *Sphingopyxis soli* Q5, *Pseudomonas aeruginosa* Q6, *Ensifer numidicus* Q7, *Nocardioides kongjuensis* Q8, *Microbacterium arborescens* Q9, *Enterobacter bugandensis* Q10, *Achromobacter mucicolens* Q11, *Olivibacter jilunii* Q12, *Aeromonas media* Q13, *Bosea eneae* Q14, *Arthrobacter.sp* Q15, and *Acinetobacter pittii* Q16*.* To further evaluate the effects of the core microbes on ungrafted watermelon plants health, we combined these 16 core strains to construct a synthetic community, hereafter referred to as SynCom.

### SynCom promoted ungrafted watermelon growth and alleviated disease

**Fig. 1:**
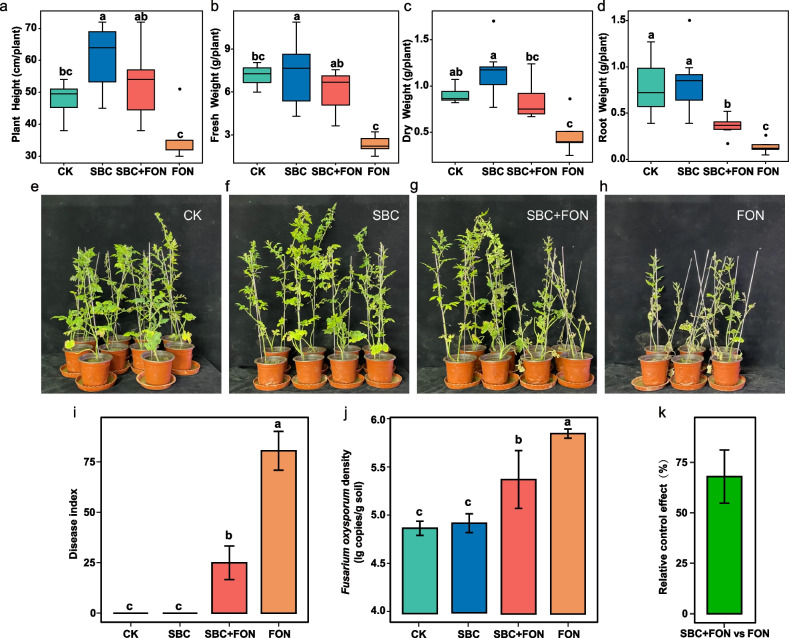
The consortium promoted plant growth and alleviated disease. **a**–**d** Shoot height, fresh weight, dry weight, and root weight of the ungrafted watermelon plants. **e**–**h** Representative images of ungrafted watermelon plants at 6 weeks post-inoculation with sterile water (CK), SynCom (SBC), SynCom and *F. oxysporum* (SBC + FON), and *F. oxysporum* (FON). **i** Fusarium wilt disease index under different treatments (CK, SBC, SBC + FON, and FON). **j** *F. oxysporum* density in the rhizosphere of different treated plants. **k** Relative control effect of the inoculated SynCom. Relative control effect (%) = [(control disease index—treatment disease index) / control disease index] × 100. Here, control refers to FON group, treatment refers to SBC + FON group. Different letters above the boxes indicate significant differences at *P* < 0.05 according to the Duncan test (*n* = 8–10). CK ungrafted watermelon plants inoculated with sterile water, SBC ungrafted watermelon plants inoculated with SynCom, SBC + FON ungrafted watermelon plants inoculated with SynCom and *F. oxysporum*, FON ungrafted watermelon plants inoculated with *F. oxysporum*

We designed four different treatment groups under greenhouse condition (in non-sterile potting soil) with ungrafted watermelon plants inoculated with either sterile water (CK), SynCom (SBC), *F. oxysporum* (FON), and both SynCom and *F. oxysporum* (SBC + FON). Our results indicated that the SBC groups exhibited significantly better growth than the CK and FON groups, significantly higher height and fresh weight values than the CK group, and 29% greater dry weight than the CK group (Fig. 1). The plant height, fresh weight, and root weight in the SBC + FON group were significantly higher than the FON group (Fig. 1a–d). Further, the SBC + FON group conferred pronounced disease resistance on ungrafted watermelon plants compared to the FON group (Fig. 1g, h). Specifically, the SBC + FON group exhibited significantly lower disease index (with a 55.6% reduction) and *F. oxysporum* abundance (with an 8.4% reduction) than the FON group (Fig. 1g–j). When comparing the SBC + FON group with the FON group, the relative control effect reached 68.0% (Fig. 1k). In addition, 11 strains (targeted ASVs) in the SynCom were able to successfully colonize roots under inoculated SynCom conditions (Fig. S7; Supplementary Methods 1.9 and 2.1). While the other five members of SynCom are not shown because they fell below the filtering criteria (Fig. S8). Overall, SynCom inoculation led to enhanced plant growth and disease resistance.

### Variation in rhizosphere bacterial community characteristics post-SynCom inoculation

**Fig. 2:**
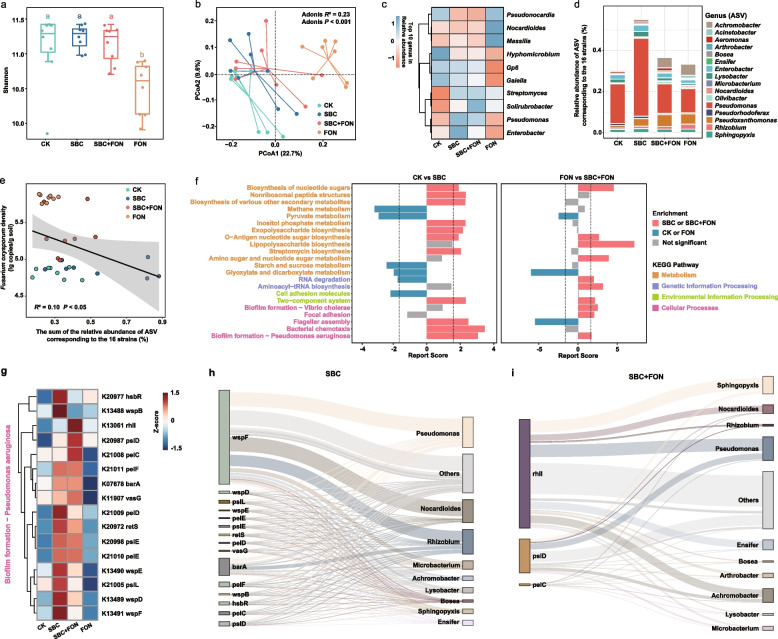
Changes in the rhizosphere microbial community composition and function profile in plants inoculated with SynCom. **a** The bacterial α diversity of CK, SBC, SBC + FON, and FON groups (*n* = 8 biologically independent plants). **b** Bray–Curtis similarity analysis of bacterial communities. **c** Bacterial relative abundance in each treated group at the genus level (top 10). **d** Relative abundances of the core microbes ASVs in the rhizosphere of different treated group. Matched V4–V5 subregion of the strain 16 S rRNA gene to ASVs as an indicator of strain presence and relative abundance in the rhizosphere (Table S4). **e** Correlation analysis of the sum of relative abundances of ASVs corresponding to each of the 16 strains with the density of *Fusarium oxysporum* in each treated group. **f** Reporter score bar plot comparing the abundance of KEGG pathways in different treated group (*n* = 3). Dashed lines represent a reporter score of 1.69, which is the threshold for indicating significant differences in such analyses. The red bars show the metabolic pathways that were significantly enriched in the SBC or SBC + FON groups. The blue bars show the metabolic pathways that were significantly enriched in the CK or FON groups. **g** Differences in the abundance (TPM) of the functional genes that are involved in biofilm formation—*Pseudomonas aeruginosa* pathway. The gene abundance shown here is Z-score standardized. **h** Genes significantly enriched in the biofilm formation—*Pseudomonas aeruginosa* pathway in the SBC group traced back to species taxonomy using meta-linking methods. **i** Genes significantly enriched in the biofilm formation—*Pseudomonas aeruginosa* pathway in the SBC + FON group traced back to species taxonomy

To elucidate the potential mechanism of action underlying the protective effects of the SynCom, the rhizosphere microbial community compositions and metagenomic profiles of the four treatment groups were compared. We observed a significantly lower microbial diversity in the FON group than the other treatment groups (Fig. 2a); however, the diversity in the SBC + FON group was comparable to that in the CK and SBC group, indicating that SynCom inoculation could normalize microbial diversity in response to pathogen infestation. The PCoA using Bray–Curtis distance showed a significant difference between microbial profiles of SBC + FON and FON group (Fig. 2b). Genus level annotation revealed an increase of the relative abundances of *Pseudomonas*, *Enterobacter*, *Solirubrobacter*, and *Streptomyces* members post-SynCom inoculation (Fig. 2c). The sum of the relative abundances of the 16 strains belonging to these targeted ASVs was higher in the SBC and SBC + FON groups than the CK and FON groups (Fig. 2d), and the sum of the relative abundance of ASVs corresponding to the 16 strains was significantly negatively correlated with pathogen abundance (Fig. 2e). Apart from variations in microbial diversity and community composition, we also observed variations in the co-occurrence networks within the microbiomes of the different groups. The microbiome network of SBC and SBC + FON groups were much more complex than the CK and FON groups, respectively, with a greater number of nodes and edges, a higher degree of modularity (Fig. S9a-f), and improved network stability (e.g., robustness, connectivity) (Fig. S9h-i). Inoculating with the SynCom also increased the positive cohesion of the community to some extent (Fig. S9g), potentially promoting community microbial cooperation that facilitates disease resistance (Fig. S10).

### Watermelon rhizosphere community function responses triggered by SynCom

We used metagenomics to determine the functional properties of the rhizosphere community that are triggered by the SynCom. Our results showed that the functional compositions of the microbiomes of the SBC and SBC + FON groups were significantly different from those of the CK and FON groups (Fig. S11a-c). To further characterize the microbial functions, we first annotated all genes to the KEGG database. We then combined the reporter feature algorithm with the KEGG metabolic networks, pathway annotations, and relative gene abundance (TPM) and found significant differences in KEGG pathway abundance between CK and SBC groups as well as between FON and SBC + FON groups. Remarkably, same pathways were enriched for the SBC and SBC + FON groups, including the two-component system, biofilm formation, etc. (Fig. 2f). SynCom-treated (SBC and SBC + FON) groups also significantly increased the abundance of the modules involved in RNA processing and modification and cell motility (Fig. S11d).

Notably, SynCom inoculation was found to substantially increase the abundance of rhizosphere *Pseudomonas* (Fig. 2c). Among the SynCom members, *Pseudomonas* belonging to the targeted ASV 37 demonstrated an effective ability to colonize the plant root system (Fig. S7). Correlation analyses unveiled a significant negative correlation between the relative abundances of *Pseudomonas* (ASV 37) and the abundance of rhizosphere pathogens (Fig. S12). Moreover, our metagenomics analysis highlighted a significant enrichment of the *Pseudomonas* biofilm synthesis pathway in SynCom-treated groups. These findings indicated that *Pseudomonas* displayed a particularly striking pattern closely associated with SynCom inoculation treatments and the impact on plant diseases. Therefore, we identified *Pseudomonas* as pivotal in enhancing plant health, prompting us to conduct a more detailed examination of this taxon.

Our next focus is on examining the changes induced by SynCom inoculation at the gene level within the *Pseudomonas* biofilm formation pathway. Metagenomic analysis revealed a total of 16 significantly enriched genes in the *Pseudomonas* biofilm formation pathway in the SBC and SBC + FON groups compared to the CK and FON groups (Fig. 2g). Most of these genes were associated with c-di-GMP signaling pathway, Gac/Rsm pathway, and Psl polysaccharide biosynthesis, which significantly influence bacterial motility and biofilm formation [43, 44]. To determine the specific bacterial taxa that might play a key role in the gene enrichment process, we further identified the species taxonomic origin of these significantly enriched genes. The flow plots indicated that these enriched genes primarily originated from members of the SynCom (Fig. 2h-i). Overall, these findings suggested that *Pseudomonas* plays a key role in disease suppression and growth promotion in watermelon plants and that other members of the SynCom drive the enrichment of *Pseudomonas* biofilm formation pathway.

### Potential interactions among members of SynCom

Based on the above amplicon and metagenomics analyses, we hypothesized that the synergistic interactions between SynCom members and *Pseudomonas* contribute to the growth and colonization of *Pseudomonas* within the rhizosphere system, ultimately promoting plant growth. To further validate the hypothesis, we employed an in vitro co-culture system to assess the synergistic effects of SynCom strains on *Pseudomonas*.

**Fig. 3:**
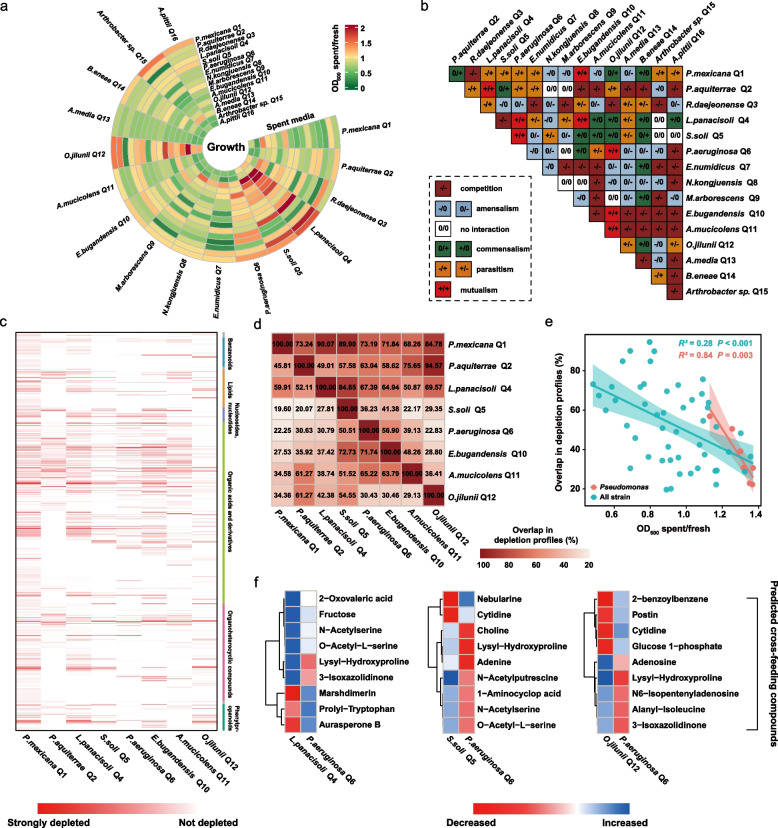
In vitro interaction matrix and substrate depletion profiles between individual SynCom strains. **a** The mean OD_600_ of the cultures of different strains in fresh medium and the respective spent medium (SM) was determined from three independent experiments. OD_600_ spent/fresh values of > 1 and < 1 indicated promotion and inhibition of the strain growth, respectively. **b** Using the OD_600_ spent/fresh values, a pairwise interaction matrix was generated. Interactions where the ratio was significantly > 1 (*P* < 0.05) are indicated with ( +), interactions where it was significantly < 1 are indicated with ( −), and interactions where the ratio did not significantly vary from 1 are indicated with (0). **c** Depletion profiles of substrates after bacterial growth to stationary phase in the fresh medium were determined by untargeted MS from three independent experiments. Metabolomic features (rows) that significantly decreased (*P* < 0.05 compared to fresh media) compared to fresh medium for the 16 strains are shown in red. Dark red indicates strong depletion, while white indicates no depletion of the metabolomics feature. **d** Pairwise overlap in depleted metabolomic features relative to the total number of depleted metabolomic features (shown in Fig. S15) of every individual strain, e.g., *P. mexicana* Q1 shares 208 metabolomic features from its set of 454 depleted metabolomic features with *P. aquiterrae* Q2, corresponding to 45.81%. As *P. aquiterrae* Q2 in contrast only depletes 284 metabolomic features in total from fresh medium, this corresponds to an overlap of 73.24% of shared metabolites between *P. aquiterrae* Q2 and *P. mexicana* Q1 relative to the total set of *P. aquiterrae* Q2 depleted metabolomics features. **e** Correlation analysis of OD_600_ spent/fresh and the pairwise overlap in depletion profiles. The blue dots represent growth involving all strains, the red dots only represent the growth of *P. aeruginosa* Q6 in the SM of the other seven strains. **f** Potential cross-feeding metabolites were identified by comparing the SM metabolic profiles (determined by untargeted MS) of the two strains. The blue color represents that one of the strains produces the metabolite and the red color represents that its partner can consume the metabolite

We primarily observed positive correlation among these core microbes in the co-occurrence network (Fig. S13). Then, analysis of the competitive potential of these core strains, which was determined by calculating their functional distance (Fig. S14; see Supplementary Methods 1.10), revealed low levels of competition among them. To further determine the interactions among these core strains, we used supernatant assays and metabolomics analyses. In detail, we cultured each strain in the sterile spent culture medium (SM) of other SynCom members to assess the interactions among SynCom members mediated by the waste products or bacteriocins in each SM. Our results showed that the SM of *P. mexicana* Q1, *P. aquiterrae* Q2, and *B. eneae* Q14 strongly promoted the growth of ~ 60% of the SynCom strains (OD_600_ spent/fresh > 1) (Fig. 3a).

To identify directionality and modes of the interactions within the SynCom strains, we constructed an interaction matrix based on OD_600_ spent/fresh values. The matrix primarily categorized the interactions among the strains as competitive (− / − , 34 out of 120 interactions) or amensalistic (0/ − or − /0, 30 out of 120 interactions) (Fig. 3b). We identified that seven strains enhanced *Pseudomonas aeruginosa* Q6 (*P. aeruginosa* Q6) growth (i.e., those exhibiting a positive interaction ( +) with *P. aeruginosa* Q6). Among these, three strains engaged in mutualistic interactions (+ / +) with *P. aeruginosa* Q6 (Fig. 3b). To investigate whether metabolic facilitation drove synergistic cooperation between *P. aeruginosa* Q6 and these seven strains, we conducted a metabolomic analysis of the SM of these eight strains, which revealed variable metabolomic features of these SM (Fig. 3c). In addition, assessment of the overlap in substrate consumption profiles (Fig. 3d) showed that *P. aeruginosa* Q6 exhibited less overlap with the other seven strains. Correlating the growth promotion in SM (OD_600_ spent/fresh) with the pairwise overlap in depletion profiles (Fig. 3d) revealed that a smaller overlap is correlated with a stronger growth promotion in the corresponding SM (*R*^2^ = 0.28, *P* < 0.001, Fig. 3e). Notably, *P. aeruginosa* Q6 had a lower degree of substrate depletion overlap with the other seven strains and displayed strong synergistic growth effects (OD_600_ spent/fresh > 1, red dots) in vitro (*R*^2^ = 0.84, *P* = 0.003, Fig. 3e). To explore potential cross-feeding metabolites between *P. aeruginosa* Q6 and the three strains showing mutualistic relationships, we delved into the metabolomic data of the SM for features enriched (or depleted) in these strains. We identified compounds such as n-acetyl serine and lysine-hydroxyproline (Fig. 3f) in the SM of these strains, indicating metabolic cross-feeding potential among these species. Overall, these results clearly indicated that the growth promotion between strains is primarily attributed to the smaller overlap of substrate consumption profiles.

### Performance of the eight species consortia in the rhizosphere

**Fig. 4:**
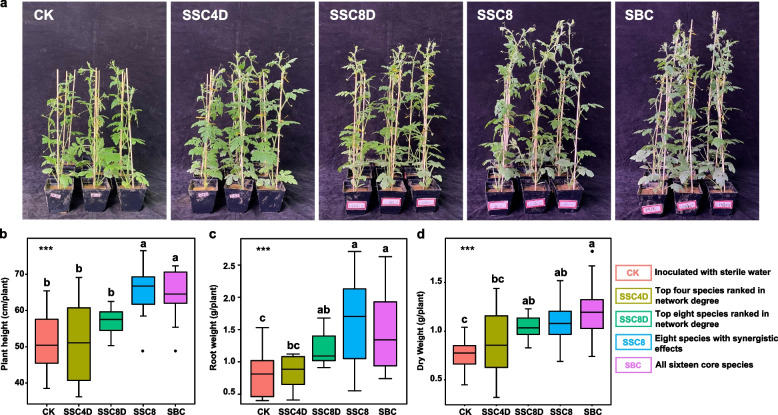
Effects of various simplified SynComs on plant growth. **a** Representative images of ungrafted watermelon plants at 6 weeks post-inoculation with sterile water (CK), top 4 species ranked in network degree (SSC4D), top 8 species ranked in network degree (SSC8D), 8 species with synergistic effect (SSC8), and all 16 core strains (SBC). **b–d** Shoot height, root weight, and dry weight of plants. Letters indicate significant differences (*P* < 0.05)

We further examined the growth-promoting effects of the simplified SynCom comprising eight bacterial strains (*P. aeruginosa* Q6 and the seven strains with synergistic effects on it, referred to as SSC8) in ungrafted watermelon plants. We observed that compared to CK, the plants of SSC8 group exhibited significantly enhanced growth (Fig. 4a). Notably, the growth parameters of the SSC8 group plants, including plant height, root weight, and dry weight, were comparable to the initial SynCom (SBC) group (Fig. 4b–d). However, when *P. aeruginosa* Q6 was combined with SynCom other strains (SSC4D and SSC8D groups), their growth-promoting effect was notably weaker than the SBC group. These results support the hypothesis that the simplified SynCom could induce beneficial effects via the synergistic interactions among the microbes present in it, thereby promoting plant health to the same extent as the initial SynCom.

## Discussion

Previous research and experiences have shown that the continuous cropping of ungrafted watermelon plants led to severe continuous cropping obstacles, resulting in inhibited plant growth and eventual withering and death [8]. Conversely, grafted watermelon plants have been shown to consistently exhibited healthy growth. Moreover, grafted watermelon plants reportedly thrive and maintain healthy growth even when cultivated in soil that has previously undergone continuous cropping of ungrafted watermelon. Notably, plant rhizosphere microbiota plays an important role in plant growth, health, and pathogen resistance [45–48]. Therefore, we focused on the SynComs derived from the rhizosphere microbiota of grafted watermelon plants to understand their role in protecting the health of ungrafted watermelon plants and underlying interaction mechanisms.

In our study, we observed that in a highly diverse grafted plants rhizosphere microbial community, the abundance of pathogens is significantly reduced, suggesting that disease suppression is a result of collective microbial interactions rather than the actions of a single species. We also identified several well-known potential biocontrol taxa, such as *Streptomyces* [20] and *Rhizobium* [49], enriched in grafted plants compared to ungrafted plants. These results indicated that grafted watermelon plants alter the composition of rhizosphere microbial communities, and these communities play a crucial role in maintaining plant health and suppressing fusarium wilt disease by recruiting specific microorganisms [50–52]. Importantly, the rhizosphere microbial community of the grafted watermelon plants could serve as a valuable resource for agricultural biocontrol, expanding the pool of cultivable microbes with potential biocontrol applications.

Deciphering the core taxa and their correlations with the host plant and disease-causing pathogens is critical for harnessing the plant microbiome to enhance plant growth and health [3, 12, 20]. However, it is unclear which core microorganisms in the grafted watermelon rhizosphere to perform specific functions. A growing body of research suggests that the core microbes can be defined as high-occupancy taxa that persist across various habitats or environments [15, 53]. From an ecological perspective, generalists are those taxa that inhabit various environments or environmental gradients [17], demonstrating high occupancy or niche width and persisting across multiple assemblages associated with a habitat. Therefore, most of these generalist species are core members of the microbial community [15, 16]. To identify microbial generalists, isolated strains were mixed in equal volumes and inoculated into different habitats (nine soils with varying fertility gradients). It is evident that soils with different fertility gradients exhibit distinct variations in physical and chemical properties. Significant differences were also observed in bacterial community composition among the different habitats. These findings further strengthen the idea that the soil fertility gradients as strong environmental filters, enabling the establishment of distinct microbial communities in the different fertility gradient soils. Consequently, each soil with a fertility gradient was considered a potential habitat. By analyzing the ecological niche distribution of cultivable microorganisms in different habitats, we selected core microbes to construct a suitable microbial combination, thereby enhancing the assembly efficiency of disease-resistant microbial communities.

The rhizosphere bacterial communities of the SynCom-inoculated plants (SBC and SBC + FON groups) exhibited notably higher relative abundances of *Enterobacter*, *Pseudomonas*, and *Solirubrobacter* than the CK and FON groups. Many strains within these genera have previously been documented to enhance plant growth and bolster plant resistance against diseases [54, 55]. Despite our study also indicated that inoculation with SynCom increased the complexity and stability of plant rhizosphere microbial networks, microbial community data is compositional [56], and the use of the Spearman correlation to construct a co-association network on compositional data can lead to the identification of spurious correlations [57].

Furthermore, the colonization of microorganisms in the root or rhizosphere is essential to control the incidence of soil-borne diseases and serves as a common indicator of the importance of microbial strains. A popular approach involves assessing the read counts of microbial sequences to estimate the presence or colonization level of microbes within a natural microbiome [17]. In line with this approach, the 11 members of the SynCom were enriched and colonized in the rhizosphere of plants in the SBC and SBC + FON groups. The correlation between the abundance of an individual strain and its ability to trigger a specific effect, such as enhanced disease resistance, reflects the relative importance of the strain [58]. We extended this concept and approach to SynCom, and correlation analysis was used to further validate the effect of SynCom abundance (the sum of the relative abundance of ASV corresponding to the 16 strains) on plant disease resistance and the importance of core microbes. Our data also revealed significant changes in the functional compositions of rhizosphere bacteria following SynCom inoculation, particularly showing an increase in the abundance of pathways such as biofilm formation. Biofilm formation aid plants in nutrient absorption and act as a biocontrol mechanism to combat diseases [59]. For example, downy mildew infection in *Arabidopsis* triggers the formation of a three-bacterial species consortium that synergistically interacts and promotes biofilm formation, reducing disease incidence [46]. Additionally, plant growth-promoting rhizobacteria cooperatively interact with native bacteria via cross-feeding, thereby synergizing biofilm formation and enhancing plant growth-promoting and salt stress-relieving ability [29]. Our results highlighted that the expression of significantly enriched pathways plays a crucial role in assisting watermelon plants in resisting disease and promoting growth. Consequently, the application of SynCom is a promising method for RMT, particularly showing significant potential in controlling soil-borne diseases [10].

We further observed that SynCom inoculation led to an increase in the relative abundance of *Pseudomonas* at the rhizosphere, particularly a specific dominant ASV 37, which exhibited the strongest response and was negatively correlated with the abundance of *F. oxysporum*. *Pseudomonas* exhibits multiple potential beneficial effects in supporting plant health, including antibiotics production and activation of induced systemic resistance [60–63]. For example, *Pseudomonas* have demonstrated remarkable biocontrol efficacy against soil-borne disease like Fusarium wilt by inducing systemic resistance in plants [60]. Previous researches have indicated that specific microbiota associated with the suppression of plant diseases (e.g., *Pseudomonas*) may be influenced by external stimuli, such as the application of bio-organic fertilizers [47] or the SynCom inoculation [26]. Stimulating the activity of these soil microbiota could particularly enhance plant disease suppression. Interestingly, we discovered that SynCom inoculation led to significant enrichment of the *Pseudomonas* biofilm formation pathway. Cooperative biofilm formation is a common trait among beneficial plant-associated microorganisms [45, 46]. Root-associated bacteria typically inhabit multi-species biofilms, and this lifestyle promotes the emergence of several characteristics within the symbiotic community, such as increased antibiotic resistance, horizontal gene transfer, and sharing of common metabolic products [64]. Furthermore, microbial positive interactions can enhance biofilm formation and microbial colonization in the rhizosphere, subsequently improving plant health [65–68]. These disease suppression or growth promotion effects are often attributed to the collective actions of the bacterial populations, while the action of individual species lead to weaker outcomes [69, 70]. Hence, it is possible that SynCom inoculation further enhances the colonization of *Pseudomonas* in the rhizosphere via interspecific synergistic effects, leading to effective growth promotion [26, 47]. Although this study has now elucidated the function and relevance of SynCom, their mechanisms of protection and cooperation still require individual and collective validation via in vitro culture-based methods.

Interestingly, the core microbes selected in the present study showed more positive co-occurrence patterns than other members of the cultivable microbes, indicating non-competitive relationships driving the coexistence of the core bacteria. This might be attributed to niche partitioning, which reduces their competitive pressure and allows them to coexist [71]. This suggestion is supported by our functional distance calculation results. We further investigated pairwise interactions among the SynCom members using supernatant culture experiments. Notably, seven strains had a promoting effect on *P. aeruginosa* Q6, possibly because these strains have different consumption profiles, which may lead to minimized competition for resources, allowing them to coexist and even be mutually beneficial [72]. These results indicated that the smaller the overlap of metabolic consumption profiles between two bacterial species, the more they tend to cooperate with each other, such as facilitating metabolic cross-feeding [73]. Synergistic interactions mediated by nutrient exchange contribute to the stability of the bacterial consortium, ensuring the optimal functions of the consortium [21, 26]. Finally, we verified whether the simplified SynCom (SSC8), comprising *P. aeruginosa* Q6 and the seven species positively interacted with it, could enhance plant health. We observed that SSC8 induced a similar plant growth promotion as the initial SynCom (SBC). Although the other simplified SynCom (SSC4D and SSC8D groups) contained *P. aeruginosa* Q6, not all other members of these two treatments were synergistic with *Pseudomonas*. These two simplified SynCom also did not exhibit a noticeable growth-promoting effect. These results suggest that the application of the SynCom comprising *Pseudomonas* and other strains that synergize with *Pseudomonas* provides a synergistic effect on plant health, including promoting plant growth. This finding underscored the importance of beneficial core microorganisms in enhancing plant health, particularly in the context of positive interactions with *Pseudomonas* [26, 74]. Inspired by the colonizing effect of SynCom members in plant roots and the positive interactions in vitro, we suggest that SynCom members can act as pioneers, occupying the available ecological niches in the rhizosphere during the plant early growth stages [75, 76]. Through metabolism, the metabolites they secrete can enhance the vitality of *Pseudomonas* within the SynCom and successor *Pseudomonas* [26]. In line with this suggestion, the metabolites (SM) of the other seven strains can promote *Pseudomonas* growth. In agreement with the metabolomic data, *P. aeruginosa* Q6 had a stronger ability to utilize organic acids, some strains can secrete organic acids which could be metabolized by *P. aeruginosa* Q6. Therefore, the optimal strain combination such as more stable or more cooperative rather than only more strains should be emphasized when constructing a SynCom [46, 77].

## Conclusions

Here, we reported that the core bacteria residing in the rhizosphere of grafted watermelon plants act as a SynCom that can effectively control disease and promote growth of ungrafted plants. SynCom inoculation was found to significantly increase the relative abundance of *Pseudomonas* in the rhizosphere and enhance the *Pseudomonas* biofilm formation pathway, suggesting that *Pseudomonas* play a pivotal role in plant health and disease resistance. In vitro assays demonstrated metabolic facilitation between some SynCom members and *Pseudomonas* improving *Pseudomonas* adaptability. Based on the interspecific synergistic effects of the SynCom members, a simplified SynCom was constructed with eight core microbes. Importantly, this streamlined version retained the growth-promoting effects observed in the original SynCom. Our research highlights that constructing and simplifying SynCom is an effective strategy for protecting plants grown in natural soil from biotic stresses. This study presents an ecological approach to promote plant health using microbial inoculants with synergistic effects, showcasing the importance of understanding and manipulating microbial interactions in the rhizosphere.

### Supplementary Information

**Additional file 1.** Supplementary Methods, Table and Figures.

**Additional file 2: Table S1.** Characteristics of bacteria in the synthetic communities.

**Additional file 3: Table S4.** Matched V4-V5 subregion of the strain 16S rRNA gene to ASVs as an indicator of strain presence and relative abundance.

## Data Availability

All raw data used in this study are available in the NCBI Sequence Read Archive (SRA), accession numbers PRJNA1001274. All pure strains were deposited in the Jiangsu Agricultural Microbial Germplasm Resources Collection, as detailed in the accession number in Table S1.
