# Supplementary material for: Synthetic community derived from grafted watermelon rhizosphere provides protection for ungrafted watermelon against Fusarium oxysporum via microbial synergistic effects
Source: Microbiome. 2024 Jun 5;12:101. doi: 10.1186/s40168-024-01814-z (PMC11151650; doi:10.1186/s40168-024-01814-z)
Supplement: Supplementary file 2 — Additional file 1. Supplementary Methods, Table and Figures. [file 40168_2024_1814_MOESM1_ESM.docx]

**Synthetic community derived from grafted-watermelon rhizosphere provides protection for ungrafted-watermelon against Fusarium oxysporum via microbial synergistic effects**

Yizhu Qiao ^a^, Zhendong Wang ^a^, Hong Sun ^a^, Hanyue Guo ^a^, Yang Song ^c^, He Zhang ^a^, Yang Ruan ^a^, Qicheng Xu ^a, b^, Qiwei Huang ^a^, Qirong Shen ^a^, Ning Ling ^a,^ ^b, *^

*a,* *Key lab of organic-based fertilizers of China and Jiangsu provincial key lab for solid organic waste utilization, Nanjing Agricultural University, Nanjing 210095, China*

*b, Centre for Grassland Microbiome, State Key Laboratory of Herbage Improvement and Grassland Agro-Ecosystems, College of Pastoral Agriculture Science and Technology, Lanzhou University, Lanzhou 730020, China*

*c,* *Plant-Microbe Interactions, Department of Biology, Science4Life, Utrecht University, Padualaan 8, 3584 CH Utrecht, the Netherlands*

**Corresponding authors:**

** Ning Ling, Email: nling@njau.edu.cn*

**1. Supplementary Methods**

**1.1 Quantitative real-time PCR amplifications (qPCR)**

The *F. oxysporum* specific primer was ITS1-F/AFR308R [1]. The assay for *F. oxysporum* was performed in a 20 µL reaction mixture containing 10 µL 2×T5 Fast qPCR Mix (SYBR Green I; TSE20, Tsingke, China), 1 µL of each primer (10 µmol/L), 1 µL of template DNA and 7 µL of sterile water. The qPCR reaction conditions consisted of pre-denaturation at 95° for 5 min, followed by 40 cycles of 95 °C for 15 s, 55 °C for 20 s, and 72 °C for 20 s. To confirm the specificity of amplification, melting curve analysis and gel electrophoresis were performed. Standard curves were used to calculate the gene copy numbers of the target group for each reaction. Each sample was analyzed in three replicates, and the results were expressed as log10 values of the gene copy numbers per gram of soil.

**1.2 Isolation and identification of culturable bacteria**

Grafted watermelon rhizosphere samples from the field were used for bacterial isolation. Rhizosphere samples were then suspended in PBS buffer (pH 7.0) and used for bacterial isolation by dilution in different culture media. The suspensions were diluted into different concentrations, and approximately 100 μL dilution was plated in different bacterial culture mediums, including tryptic soy broth (TSB) and beef extract peptone (NB) medium. The single colonies were picked based on their size, color, and morphology. Colonies were re-streaked at least twice to ensure purity. These selected single colonies were subsequently inoculated into 2 mL sterile tubes containing the corresponding medium and incubated for 5 days. Afterward, the bacterial DNA was extracted from each isolate using lysis buffer (25 mM NaOH and 0.2 mM Na_2_-EDTA, pH 12) and incubated for 30 min at 95 °C, then neutralized with the neutralization buffer (40 mM Tris-HCl, pH 7.5) [2]. Single colonies were identified based on the full-length 16S rRNA gene sequence. Briefly, the full-length of 16S rRNA gene was amplified with forward primer 27 (5’-AGAGTTTGATCMTGGCTCAG-3’) and primer 1492R (5’- TACGGYTACCTTGTTACGACTTC -3’) under the following conditions: denaturation at 95° for 4 min; 30 cycles of 95 °C for 30 s, 55 °C for 30 s, and 72 °C for 60 s, and final elongation at 72 °C for 5 min [2]. Sanger sequenced was performed at LC-Bio Technology Co., Ltd (Shanghai, China). Sequence alignment was performed on the NCBI website, and each microbial species was preserved in 30% glycerol (v/v) at −80 °C. To reveal the coverage of isolation strains in grafted watermelon rhizosphere microbiota, cultivated bacterial strains were compared with high-abundant taxa (relative abundance >0.05%) of the grafted watermelon rhizosphere bacterial ASVs.

**1.3 Experimental design of soil successions with different fertility**

Environmental conditions play a crucial role in shaping and creating distinct habitat conditions. The composition of microbial communities in these different habitats exhibits significant differences, ultimately determining the composition of core microbes. Therefore, in this study, we aimed to investigate the composition of the microbial community in different habitat conditions characterized by soil with varying fertility gradients (mixed in different proportions from high and low fertility soils) to identify the core species.

Low and high fertility soils were selected for the experiment. Low fertility soils (total carbon 4.48 g/kg, total nitrogen 1.17 g/kg, available phosphorus 30.64 mg/kg, available potassium 128.52 mg/kg, pH 7.59) were collected from watermelon planting greenhouses of Nanjing Institute of Vegetable and Flower. High fertility soil (total carbon 9.36 g/kg, total nitrogen 2.62 g/kg, available phosphorus 63.74 mg/kg, available potassium 175.66 mg/kg, pH 7.29) was collected from the rice fields of Jiangsu Academy of Agricultural Sciences. At the sites described above, soil samples were collected from the 0-20 cm depth and transported back to the laboratory for storage. A portion of the soil was subjected to air-drying to facilitate the analysis of its physical and chemical properties (Supplementary Methods 1.11). The remaining soil samples were used for the construction of soil with different fertility gradients. The construction method of 9 soil habitats with different fertility gradient is shown in Fig S5a. To be specific, high fertility soil and low fertility soil are mixed in accordance with 9 mass ratios, that is, high fertility soil (g): low fertility soil (g) = 1:9, 2:8, 3:7, 4:6, 5:5, 6:4, 7:3, 8:2, 9:1, and sequentially designated as R19, R28, R37, R46, R55, R64, R73, R82, R91. Each soil treatment contains 150 g of soil. Nine soil habitats of different fertility were obtained and three replicates were set up for each treatment. Specific mixed method is, (1) R19: 15 g of high fertility soil was mixed with 135 g of low fertility soil. (2) R28: 30 g high fertility soil mixed with 120 g low fertility soil. (3) R37: 45 g high fertility soil mixed with 105 g low fertility soil. (4) R46: 60 g high fertility soil mixed with 90 g low fertility soil. (5) R55: 75 g high fertility soil mixed with 75 g low fertility soil. (6) R64: 90 g of high fertility soil mixed with 60 g of low fertility soil. (7) R73: 105 g of high fertility soil mixed with 45 g of low fertility soil. (8) R82: 120 g of high fertility soil mixed with 30 g of low fertility soil. (9) R91: 135 g of high fertility soil mixed with 15 g of low fertility soil. The mixed soil samples were sent to the soil sterilization and irradiation center (Nanjing Xiyue Technology co., ltd) for sterilization by gamma rays. The radiation duration is 72 h and the radiation intensity is 50 kGray. To create a sterile soil habitat, mixed sterilized soil was carefully placed into a 500 mL sterile culture pot (each pot contained 150g of mixed soil). The pots were then pre-cultured for 7 days (20℃ and 45% soil field moisture capacity). After 7 days, a portion of the soil was coated on the plate. It was observed that no microorganisms grew on the plate, confirming the sterility of the created soil habitat.

The shake flask fermentation method was employed to cultivate all bacterial strains (394 strains) in NB medium for 2 days at 30 °C. Each of the bacterial fermentation broth was centrifuged at 4000×g for 5 min and re-suspended in PBS with OD_600_ adjusted to 1.0. The bacterial suspension of 394 isolated strains was mixed in equal volume (v/v), and then the 15 mL mixed bacterial suspension was added in 9 sterile soil habitats with different fertility, respectively. All of the constructed soil habitats were placed in an incubator for dark culture at 25℃ and 45% soil field moisture capacity for 90 days. At the end of the culture, soil samples were collected from each habitat (different fertility gradients). Total DNA of soil was extracted using FastDNA™ soil DNA extraction kit (MP Biomedicals, Cleveland, OH, USA), followed by 16S rRNA gene absolute quantitative sequencing (Genesky Biotechnologies, Inc., Shanghai, China).

**1.4 Absolute quantitative sequencing of 16S rRNA gene in samples of soil succession experiment with different fertility**

Total genomic DNA was extracted from different fertility soil treatment (that is, R19, R28, R37, R46, R55, R64, R73, R82, R91) and processed for 16S rRNA gene sequencing by Genesky Biotechnologies Inc. (Shanghai, China) for the relative and absolute quantification of microbiota abundance. Three replications per treatment. Briefly, the DNA samples were mixed with artificial spike-in reference sequences after quality detection and subjected to amplification of the V4-V5 variable region of the bacterial 16S rRNA gene. Sample-specific index sequences were added to the DNA pool, followed by library quantification, pooling and quality checks. Libraries were sequenced on Illumina MiSeq Sequencer (Illumina, USA) using a 2 × 250–base pair (bp) double-ended sequencing technology. Raw reads were trimmed using the tools Usearch to remove the low-quality sequences (quality score < 20), adaptor sequences, primer sequences, shorter sequences (<100 bp), and sequences with high base error rate (>2) to obtain the clean reads with high quality and credibility [3]. The remaining sequences were utilized to generate an amplicon sequencing variant (ASV) table. The spike-in DNA sequences were filtered out, and the copy number of spike-in ASV in each sample was counted. A standard curve of read count of each ASV versus spike-in DNA copy number of each sample was generated. The absolute copy number of each ASV was calculated based on the standard curve and then adjusted according to the estimated rRNA operon copy number from the rrnDB database. The final copy number was then utilized to calculate the relative abundance. The microbial community richness was analyzed using the Simpson and Shannon estimators.

**1.5 The grading of the** **disease index**

The disease severity was scored on a 5-point rating system modified from Yu et al. : 0, no disease symptoms; 1, less than 1/4 of the plant was wilted, but growth was normal; 2, the wilted part of the plant accounted for 1/4 to 1/2 of the whole plant, and the root becomes diseased; 3, more than half of the plant was wilted and root necrosis; 4, the whole plant wilted, and plant was either dead or very small and wilted [4]. Disease index = (1n_1_ + 2n_2_ + 3n_3_+ 4n_4_)·100/4N_t_, where n_1_-n_4_ is the number of watermelons plants in the indicated class and N_t_ is the total watermelon plants of all 4 classes [4]. Relative control effect (%) = [(control disease index - treatment disease index) / control disease index] × 100. Here, control refers to FON group, treatment refers to SBC+FON group.

**1.6 Bioinformatics analyses**

The bacterial communities were profiled by amplifying the V4-V5 region of the 16S rRNA gene. using the primer pairs 515 F (5’-GTGCCAGCMGCCGCGG -3’)/907 R (5’- CCGTCAATTCMTTTRAGTTT -3’).

For network analysis of inoculation treatments (Fig S9), we used a public script implementation. In constructing these networks, we used normalized ASVs tables and performed Spearman rank correlation analysis between ASVs (|ρ | > 0.6 and p < 0.05). Network parameters were calculated based on Yuan et al. 's method [5].

**1.7 Meta-links** **analysis**

Meta-links aims to correlate species and functional information by integrating information on samples with the same ORF (Open Reading frame) identifier (i.e., ORF IDs). This approach focuses on analyzing specific functional genes (using the KEGG database in this study) and aggregating the distribution of these functional genes across different species. Additionally, the approach focuses on the distribution of taxa corresponding to different genes at different taxonomic levels (e.g., genus level). The results are visualized using the Sankey diagram.

**1.8 Metabolome analysis**

LC-MS/MS analyses were performed using an UHPLC system (Vanquish, Thermo Fisher Scientific) with a UPLC BEH Amide column (2.1 mm × 100 mm, 1.7 μm) coupled to Orbitrap Exploris 120 mass spectrometer (Orbitrap MS, Thermo). The mobile phase consisted of 25 mmol/L ammonium acetate and 25 ammonia hydroxide in water（pH = 9.75）(A) and acetonitrile (B). The auto-sampler temperature was 4 ℃, and the injection volume was 2 μL. The raw data were converted to the mzXML format using ProteoWizard and processed with an in-house program, which was developed using R and based on XCMS, for peak detection, extraction, alignment, and integration. Then an in-house MS2 database (BiotreeDB) was applied in metabolite annotation. The cutoff for annotation was set at 0.3. The normalized data were used to predict the molecular formula based on additive ions, molecular ion peaks, and fragment ions. T-test was used to compare the features’ intensity from spent media with fresh media. The associated untargeted metabolomics data are available on MetaboLights repository with ID MTBLS9628.

**1.9 Colonization analysis**

To track the SynCom members in our pot experiment sequencing data, the V4-V5 subregion of the full-length 16S rRNA gene sequence of the strain were extracted and aligned with the sequences of raw ASVs as an indicator of the presence and relative abundance of the strain. The filtering criteria were that the V4-V5 region of the strain matched the ASVs by 97% or more and that the ASVs were present in at least 4 replicates [6].

**1.10 Metabolic Networks and Functional Distance**

Initially, the 16S rRNA gene sequences underwent a BLAST search against the Greengenes v13.5 16S rRNA gene database using prfectBLAST. Subsequently, the top-performing match, corresponding to a 16S rRNA gene within an Operational Taxonomic Unit (OTU) from the precomputed Kyoto Encyclopedia of Genes and Genomes (KEGG) Ortholog table provided by PICRUSt, was selected. Following this, metabolic networks were constructed from the KEGG Ortholog list using RevEcoR with specific parameters (threshold = 0, is giant = FALSE). Functional similarity between each pair was then computed by assessing the overlap of nodes (metabolites) within the two networks, divided by the average number of nodes present in both networks. The functional distance was then defined as 1 subtracted by the functional similarity. The algorithm and code are described in Russel et al. 's method [7].

**1.11 Determination of soil physical and chemical properties**

Soil pH was determined by preparing a soil suspension with a ratio of soil to distilled water of 1:2.5, which was then measured using a pH meter. Soil total nitrogen and carbon were analyzed using a carbon and nitrogen analyzer (Jena Multi EA S 5000, Germany). Soil available phosphorus was extracted with 0.5 M NaHCO_3_ and quantified using molybdenum blue colorimetry. Soil available potassium was extracted with 1 M ammonium acetate and measured using a flame photometer (M-410; Cole-Parmer, Chicago, IL, USA).

**1.12 Phylogenetic tree of** **high abundant bacterial ASVs**

The high abundant bacterial ASVs (relative abundance＞0.05%) were chosen, with 169 bacterial ASVs and associated representative sequences were used for the construction of maximum likelihood (ML) trees. The IQ-Tree software was used for the ML tree construction. The tree files were uploaded to the iTOL (http://itol.embl.de) online and the phylogenetic trees were edited, and annotated with the bar of the relative abundance of ASVs of grafted watermelon rhizosphere. Isolate bacterial strains classified into the same genera as those presented in the phylogenetic trees were included in the outer rings as pink dots.

**References**

1. Liu L, Kong J, Cui H, Zhang J, Wang F, Cai Z, Huang X. Relationships of decomposability and C/N ratio in different types of organic matter with suppression of Fusarium oxysporum and microbial communities during reductive soil disinfestation. Biological Control. 2016;101: 103-113.
2. Zhang J, Liu YX, Guo X, Qin Y, Garrido-Oter R, Schulze-Lefert P, Bai Y. High-throughput cultivation and identification of bacteria from the plant root microbiota. Nature Protocols. 2021;16: 988-1012.
3. Chen C, et al. Glucocorticoid-induced loss of beneficial gut bacterial extracellular vesicles is associated with the pathogenesis of osteonecrosis. Science Advances. 2022;8: eabg8335.
4. Yu H, Chen S, Zhang X, Zhou X, Wu F. Rhizosphere bacterial community in watermelon-wheat intercropping was more stable than in watermelon monoculture system under Fusarium oxysporum f. sp. niveum invasion. Plant and Soil. 2019;445: 369-381.
5. Yuan MM, et al. Climate warming enhances microbial network complexity and stability. Nature Climate Change. 2021;11: 343-348.
6. Schmitz L, Yan Z, Schneijderberg M, de Roij M, Pijnenburg R, Zheng Q, Cheng X. Synthetic bacterial community derived from a desert rhizosphere confers salt stress resilience to tomato in the presence of a soil microbiome. The ISME Journal. 2022; 16: 1907-1920.
7. Russel J, Røder HL, Madsen JS, Burmølle M, Sørensen SJ. Antagonism correlates with metabolic similarity in diverse bacteria. Proceedings of the National Academy of Sciences. 2017;114: 10684-10688.

**2. Supplementary Text**

**2.1 The SynCom colonized the root of ungrafted-plants under non-sterile conditions**

In microbiome studies, the primary approach to assess the significance of a strain is through the extent of root colonization. This strategy is also applicable to the investigation of interbacterial dynamics within the native microbiome or, in our specific case, the interaction between SynCom and its environment. We took the V4-V5 subregion of the 16S rRNA gene in the strain genome sequence as the expected sequence and matched it to ASVs as an indicator of strain presence and relative abundance (Table S4). This analysis showed that out of 16 strains, the ASVs of 11 strains could be detected in all treatments (Fig. S7), while the other 5 strains are not shown because they fell below the filtering criteria (Fig. S8, Supplementary Methods 1.9). Out of the 11 targeted ASVs, ASV 368 (*Pseudoxanthomonas*), ASV 422 (*Achromobacter*) and ASV 3499 (*Olivibacter*) were not detected in the control but only in the root inoculated with the SynCom. This indicates that the bacteria with these ASVs are likely not present in the non-sterile substrate and probably originated from the SynCom strains. Statistical analysis showed that the relative abundance of ASVs such as ASV 368 (*Pseudoxanthomonas*), ASV 37 (*Pseudomonas*), and ASV 422 (*Achromobacter*) was significantly increased by inoculation with SynCom compared to CK or FON group. These results suggest that the target strains belonging to these targeted ASVs can effectively establish colonization in the root system.

**Table S2 Physical and chemical properties of soils with different fertility gradients**

| Treatment | TC (g/kg) | TN (g/kg) | AP (mg/kg) | AK (mg/kg) | pH |
| --- | --- | --- | --- | --- | --- |
| R19 | 4.53±0.35 e | 1.28±0.07 f | 32.42±2.65 e | 133.67±3.51 c | 7.51±0.01 a |
| R28 | 4.65±0.10 e | 1.38±0.15 ef | 40.23±3.05 cde | 135.33±5.85 c | 7.49±0.04 ab |
| R37 | 4.95±0.24 e | 1.45±0.16 ef | 39.36±1.05 de | 137.66±10.41 c | 7.50±0.02 a |
| R46 | 5.15±0.35 e | 1.50±0.18 def | 43.09±0.30 bcde | 139.33±4.16 bc | 7.46±0.03 ab |
| R55 | 6.02±0.61 d | 1.66±0.14 cde | 49.77±7.68 abcd | 148.67±14.57 abc | 7.44±0.04 abc |
| R64 | 6.90±0.59 c | 1.78±0.07 cd | 51.94±7.14 abcd | 148.67±14.57 abc | 7.47±0.04 ab |
| R73 | 7.49±0.26 c | 1.94±0.09 bc | 52.54±3.82 abc | 160.33±2.31 ab | 7.4±0.04 bcd |
| R82 | 8.35±0.14 b | 2.17±0.20 b | 54.78±4.16 ab | 160.67±7.37 ab | 7.36±0.03 cd |
| R91 | 9.3±0.16 a | 2.58±0.04 a | 58.49±3.11 a | 165.00±7.21 a | 7.32±0.05 d |

Note: The date in the table is average value ± standard deviation，and different letters indicate significant differences at the 0.05 level. To be specific, high fertility soil and low fertility soil are mixed in accordance with 9 mass ratios, that is, high fertility soil (g): low fertility soil (g) = 1:9, 2:8, 3:7, 4:6, 5:5, 6:4, 7:3, 8:2, 9:1, and sequentially designated as R19, R28, R37, R46, R55, R64, R73, R82, R91. Methods for the determination of soil physical and chemical properties are detailed in Supplementary Method 1.11.

**Table S3 Selected core microbes of the grafted watermelon rhizosphere**

| Strain Code | Generalists ASV | Class | Order | Family | Genus |
| --- | --- | --- | --- | --- | --- |
| Q1 | 2 | Gamma-Proteobacteria | Lysobacterales | Lysobacteraceae | Pseudoxanthomonas |
| Q2 | 22 | Beta-Proteobacteria | Burkholderiales | Comamonadaceae | Pseudorhodoferax |
| Q3 | 9 | Alpha-Proteobacteria | Hyphomicrobiales | Rhizobiaceae | Rhizobium |
| Q4 | 16 | Gamma-Proteobacteria | Lysobacterales | Lysobacteraceae | Lysobacter |
| Q5 | 27 | Alpha-Proteobacteria | Sphingomonadales | Sphingomonadaceae | Sphingopyxis |
| Q6 | 7 | Gamma-Proteobacteria | Pseudomonadales | Pseudomonadaceae | Pseudomonas |
| Q7 | 39 | Alpha-Proteobacteria | Hyphomicrobiales | Rhizobiaceae | Ensifer |
| Q8 | 43 | Actinobacteria | Propionibacteriales | Nocardioidaceae | Nocardioides |
| Q9 | 42 | Actinobacteria | Micrococcales | Microbacteriaceae | Microbacterium |
| Q10 | 1 | Gamma-Proteobacteria | Enterobacterales | Enterobacteriaceae | Enterobacter |
| Q11 | 12 | Beta-Proteobacteria | Burkholderiales | Alcaligenaceae | Achromobacter |
| Q12 | 5 | Sphingobacteriia | Sphingobacteriales | Sphingobacteriaceae | Olivibacter |
| Q13 | 20 | Gamma-Proteobacteria | Aeromonadales | Aeromonadaceae | Aeromonas |
| Q14 | 38 | Alpha-Proteobacteria | Hyphomicrobiales | Boseaceae | Bosea |
| Q15 | 23 | Actinobacteria | Micrococcales | Micrococcaceae | Arthrobacter |
| Q16 | 4 | Gamma-Proteobacteria | Pseudomonadales | Moraxellaceae | Acinetobacter |

**Note:** All core microbes were isolated from grafted watermelon rhizosphere. These 16 core microbes together were used for SynCom construction.


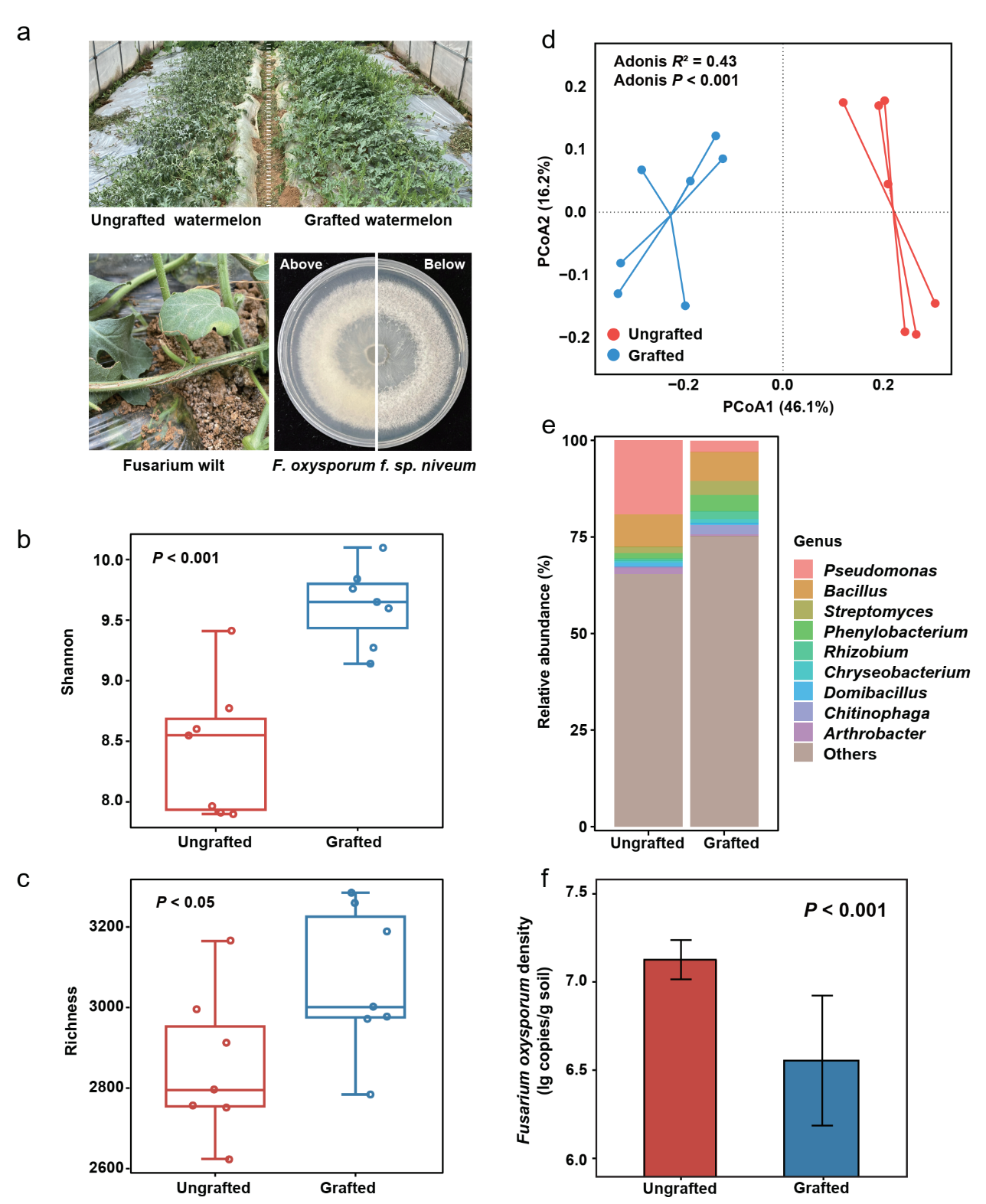
 **Fig.S1 Field images of grafted watermelon and ungrafted watermelon plants and their microbial diversity and community composition.** (a) Top row: representative sampling sites of grafted and ungrafted watermelons and their Fusarium wilt disease incidence. Bottom row: isolation of Fusarium oxysporum f. sp. niveum (*F. oxysporum*) from diseased ungrafted watermelon (left); representative colonies of *F. oxysporum* growing on PDA, photographed from above and below, respectively (right). (b) Rhizosphere bacterial Shannon diversity in grafted and ungrafted watermelon plants. (c) Rhizosphere bacterial Richness diversity in grafted and ungrafted watermelon plants. (d) Bray-Curtis similarity analysis of bacterial communities. (e) Relative abundance composition of bacterial species in the rhizosphere samples of grafted and ungrafted watermelons. (f) Comparison of *F. oxysporum* levels in grafted and ungrafted watermelon plants rhizosphere. Data bars represent means, and error bars represent the standard error of mean. The number of samples per group is 7 biologically independent plants (n=7). The horizontal line within boxes represents medians, tops and bottoms of boxes represent the 75th and 25th percentiles, and upper and lower whiskers extend to data no more than 1.5 times the interquartile range from the upper edge and lower edge of the box, respectively.


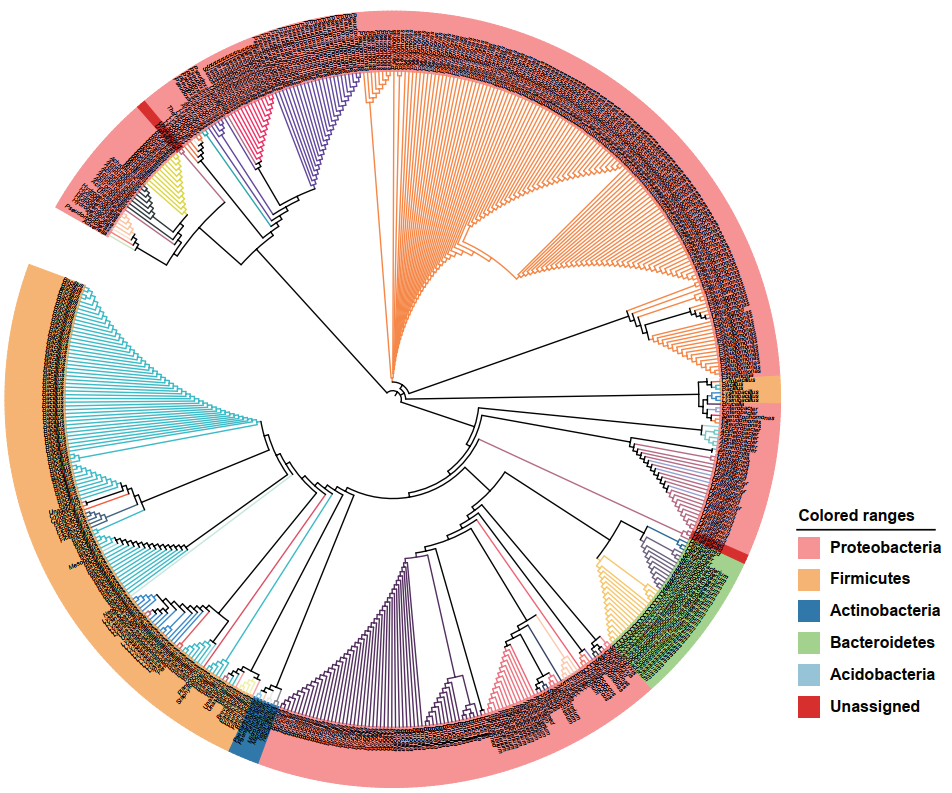


**Fig. S2 Phylogenetic tree showing the isolated and cultivated bacterial strains derived from the rhizosphere of grafted watermelon plant.** The outer ring of color blocks represents different phylum, and the branch colors represent different genus.


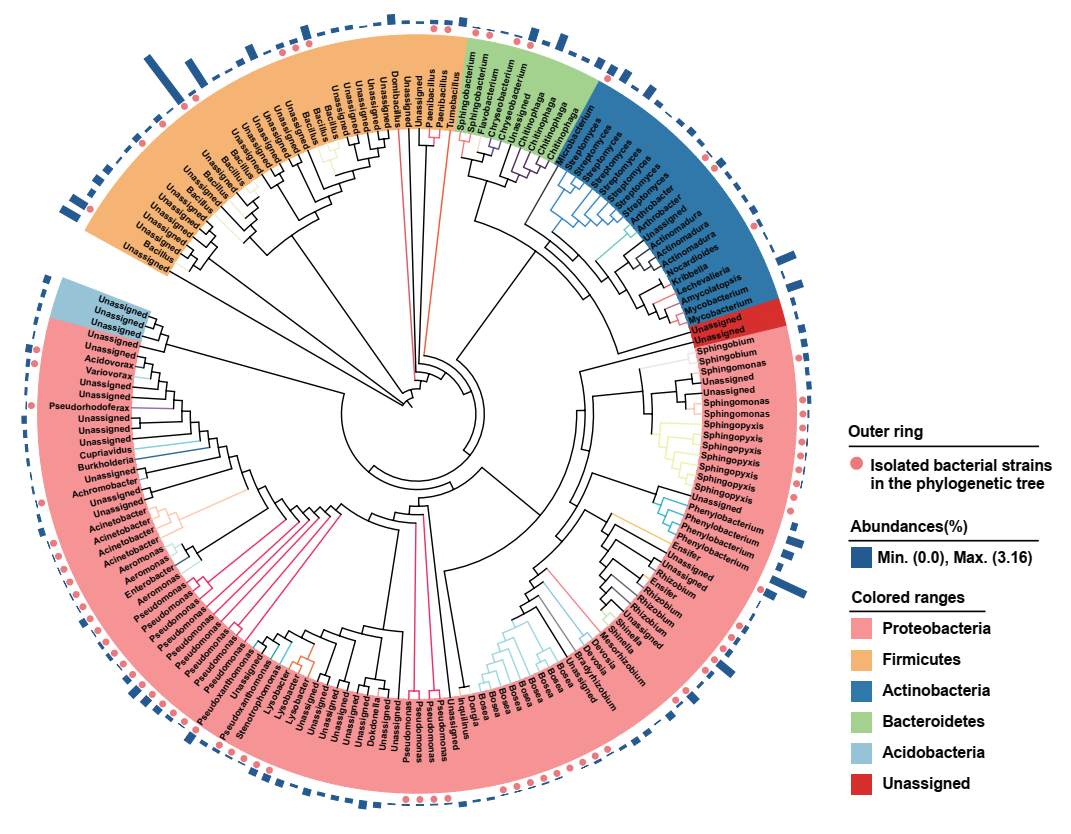
 **Fig. S3 Phylogenetic tree of high abundant bacterial ASVs (relative abundance＞0.05%).** The outer ring (bar) represents the relative abundance of each node ASVs presented at grafted watermelon plant rhizosphere. The middle ring (pink elliptical points) represents bacterial ASV identified among the isolated and cultivated bacterial strains derived from grafted watermelon plant rhizosphere. The color blocks represent different phylum, and the branch colors represent different genus. For detailed methods, see Supplementary Methods 1.12.


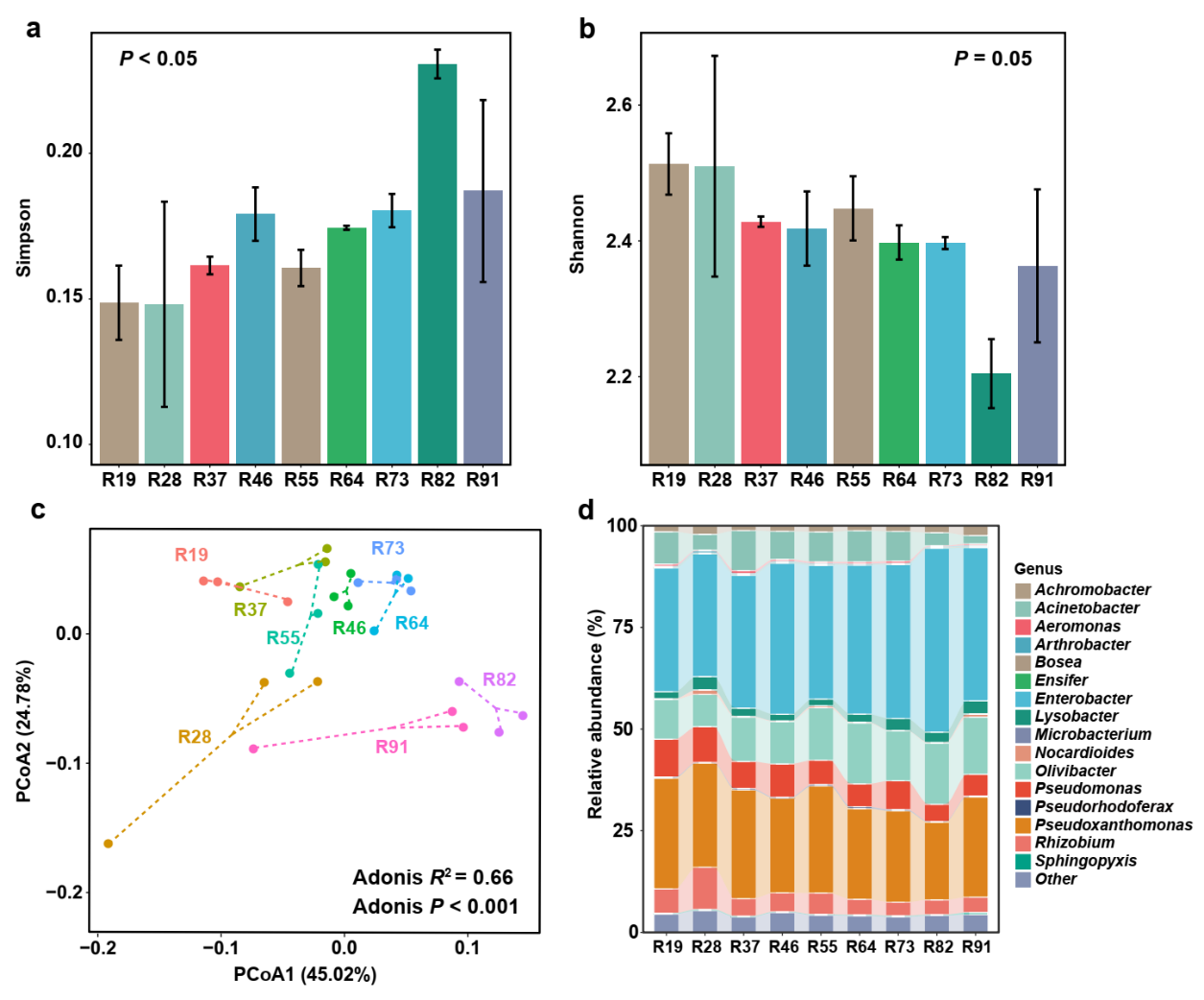


**Fig. S4 Analysis of soil community diversity and composition under different fertility gradients.** (a) Simpson diversity of soil microbial community under different fertility gradients. (b) Shannon diversity of soil microbial community under different fertility gradients. (c) PCoA analysis of soil microbial community under different fertility gradients. (d) Community composition of soil microbial genus levels under different fertility gradients. To be specific, high fertility soil and low fertility soil are mixed in accordance with 9 mass ratios, that is, high fertility soil (g): low fertility soil (g) = 1:9, 2:8, 3:7, 4:6, 5:5, 6:4, 7:3, 8:2, 9:1, and sequentially designated as R19, R28, R37, R46, R55, R64, R73, R82, R91.


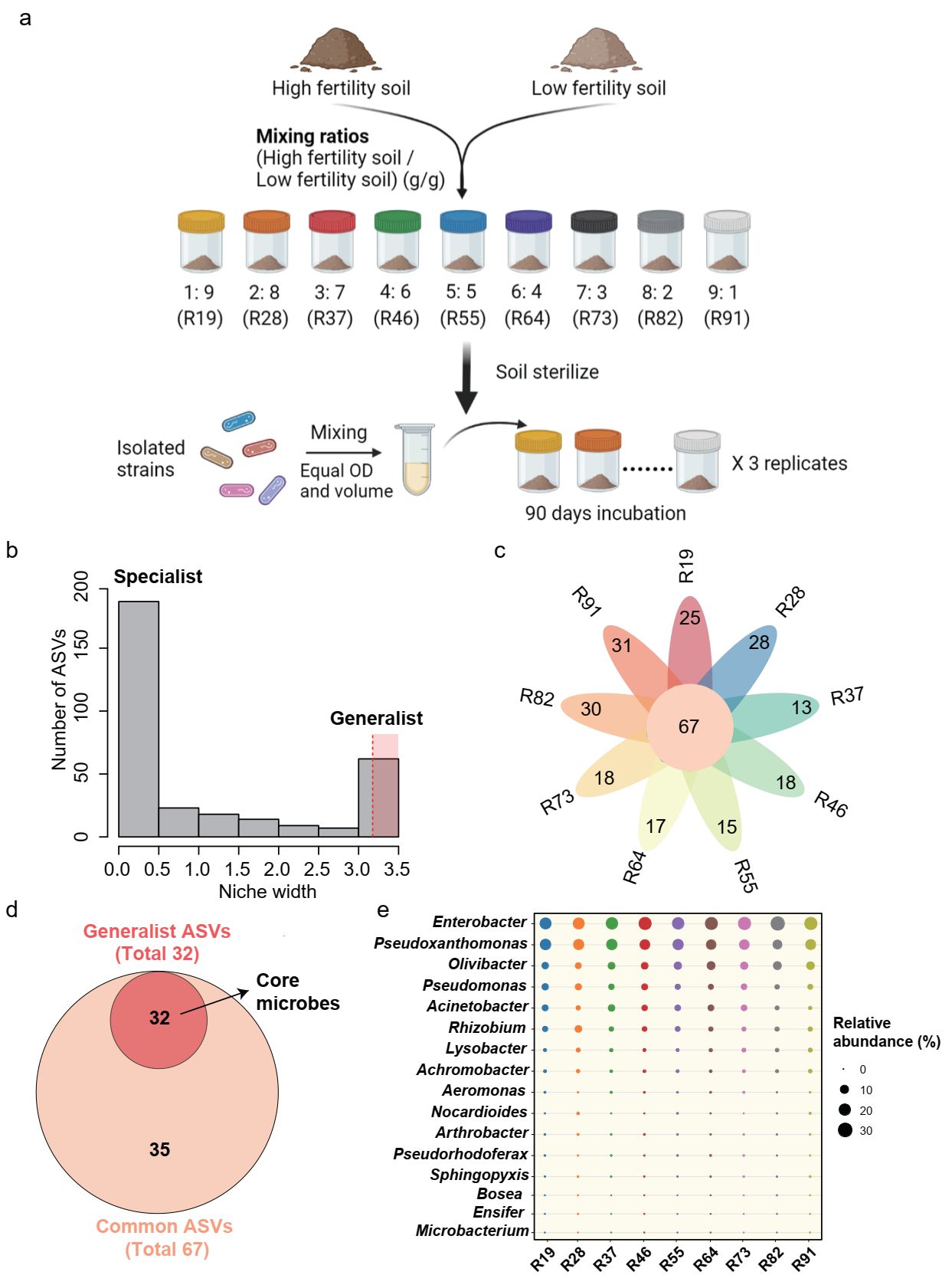
 **Fig.S5 The identification of core microbes.** (a) Experimental design of soil successions with different fertility. Soil with high and low fertility was mixed in different mixing ratios, and all the isolated bacteria were inoculated in equal amounts in each treatment. After 90 days of dark culture, DNA was extracted from soil samples. To be specific, high fertility soil and low fertility soil are mixed in accordance with 9 mass ratios, that is, high fertility soil (g): low fertility soil (g) = 1:9, 2:8, 3:7, 4:6, 5:5, 6:4, 7:3, 8:2, 9:1, and sequentially designated as R19, R28, R37, R46, R55, R64, R73, R82, R91. For detailed experimental design, see Supplementary Methods 1.3. (b) Niche width of the overall microbial communities calculated using Shannon index H′. Generalists are defined as microbes with the largest niche width (top 10th percentile), and specialists as microbes with the smallest niche width (bottom 10th percentile). (c) Venn diagram showing shared and unique ASVs between all treatments (habitats). (d) Based on our definition of core microbes as those that shared between generalists and common ASVs, we identified 16 core microbes (16 clusters of 97% similarity originating from 32 ASVs). (e) Balloon plot showing the relative abundance of the core microbes across different habitats at the genus level.

**
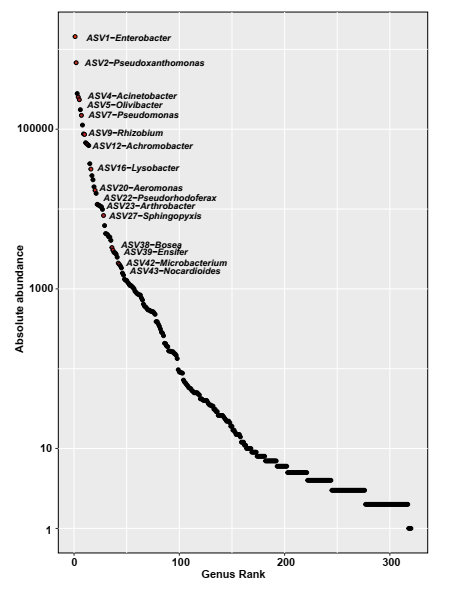
Fig. S6 Ranking of the absolute abundance of microbial communities on a logarithmic scale.**


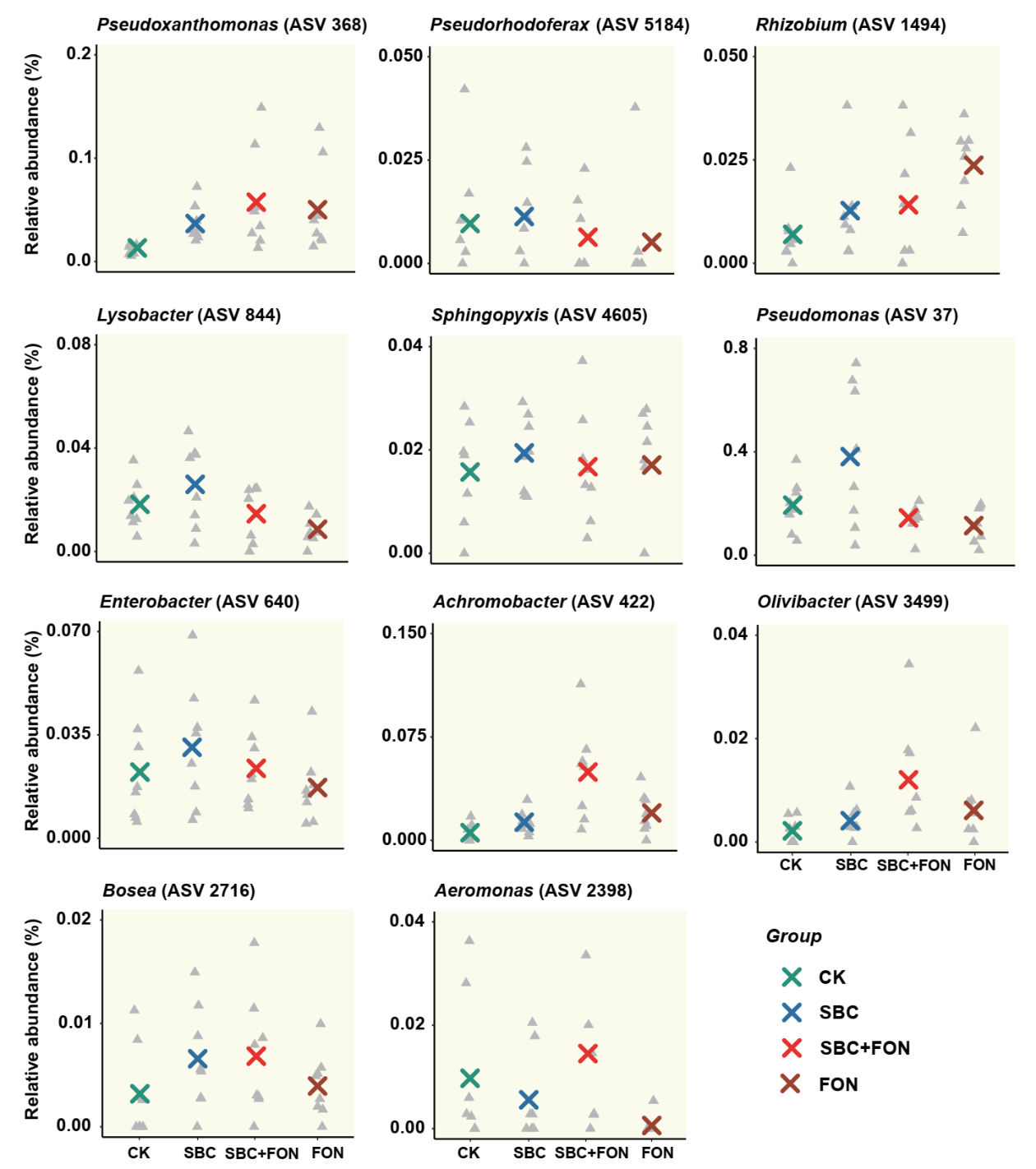


**Fig. S7** **Colonization levels of eleven members of SynCom in the rhizosphere of ungrafted watermelon plants.** These eleven ASVs exhibited a perfect match to the V4-V5 region of the SynCom strains. The relative abundances of the replicates are shaded as gray symbols per compartment and the mean is shown as a cross with the color referring to the inoculation treatment. CK: ungrafted watermelon plants inoculated with sterile water; SBC: ungrafted watermelon plants inoculated with SynCom; SBC+FON: ungrafted watermelon plants inoculated with SynCom and *F. oxysporum*; FON: ungrafted watermelon plants inoculated with *F. oxysporum*.


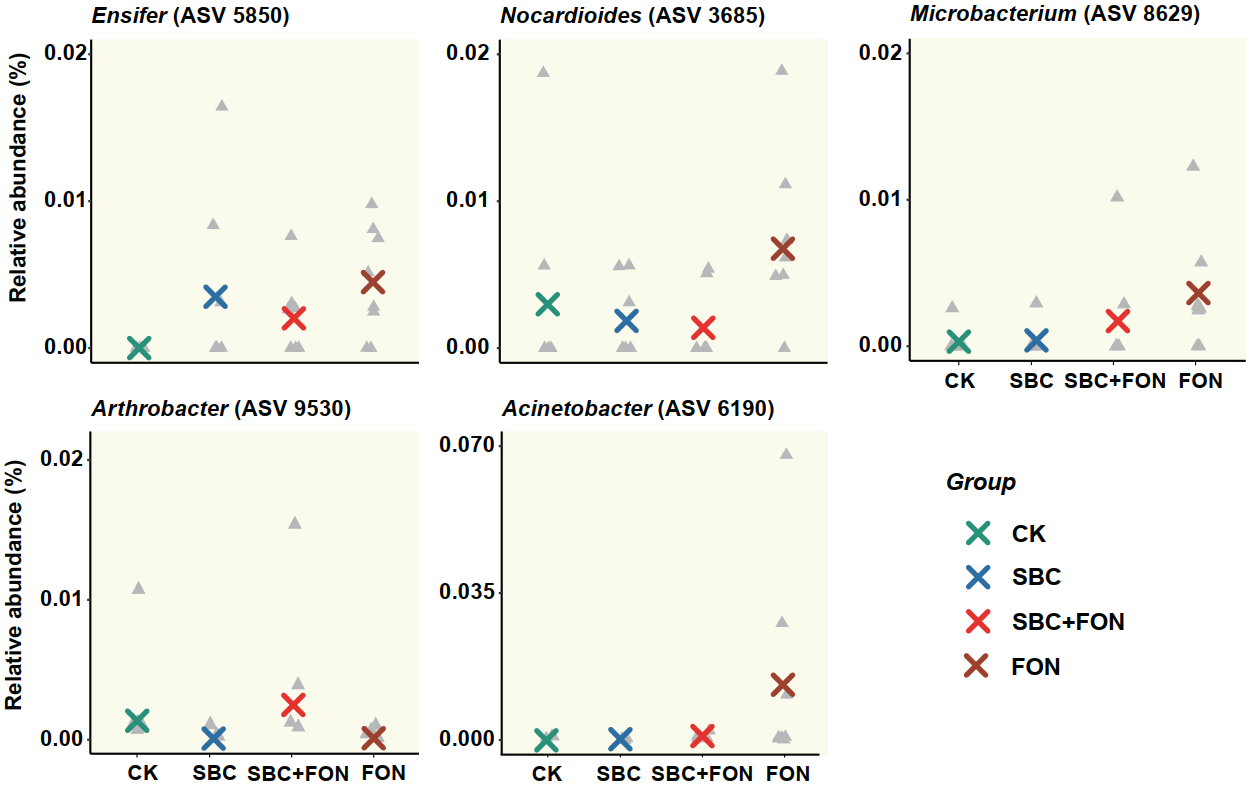
 **Fig. S8 Colonization levels of five members of SynCom in the rhizosphere of ungrafted watermelon plants.** These five ASVs exhibited a perfect match to the V4-V5 region of the SynCom strains. The five strains fell below our filtering criteria. The relative abundances of the replicates are shaded as gray symbols per compartment and the mean is shown as a cross with the color referring to the inoculation treatment. CK: ungrafted watermelon plants inoculated with sterile water; SBC: ungrafted watermelon plants inoculated with SynCom; SBC+FON: ungrafted watermelon plants inoculated with SynCom and *F. oxysporum*; FON: ungrafted watermelon plants inoculated with *F. oxysporum*.


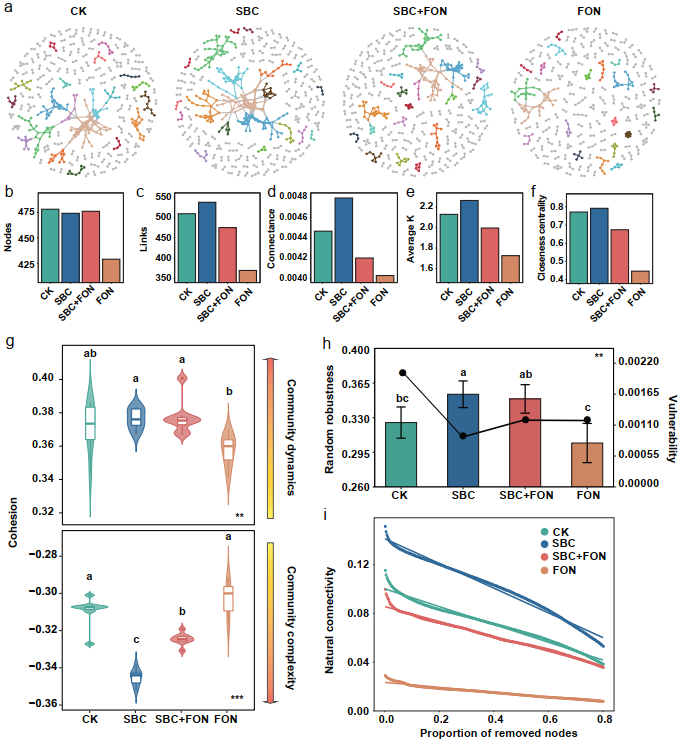


**Fig. S9 Changes of** **rhizosphere microbial community networks and their parameters under different inoculation treatments.** (a) Networks are based on Spearman’s rank correlation between the ASVs in the rhizosphere of plants growing in the non-sterile substrate with different inoculation treatments. Only edges with a correlation score |ρ | > 0.6 and a p < 0.05 are shown. Different colors represent different modules. (b-f) Differences in network parameters (nodes, links, connectance, average K, and closeness centrality) between networks based on the microbiomes of plants with different inoculation treatments. (g) Differences in network cohesion. (h) Differences in network random robustness and vulnerability. (i) Differences in network natural connectivity. CK: ungrafted watermelon plants inoculated with sterile water; SBC: ungrafted watermelon plants inoculated with SynCom; SBC+FON: ungrafted watermelon plants inoculated with SynCom and *F. oxysporum*; FON: ungrafted watermelon plants inoculated with *F. oxysporum*.


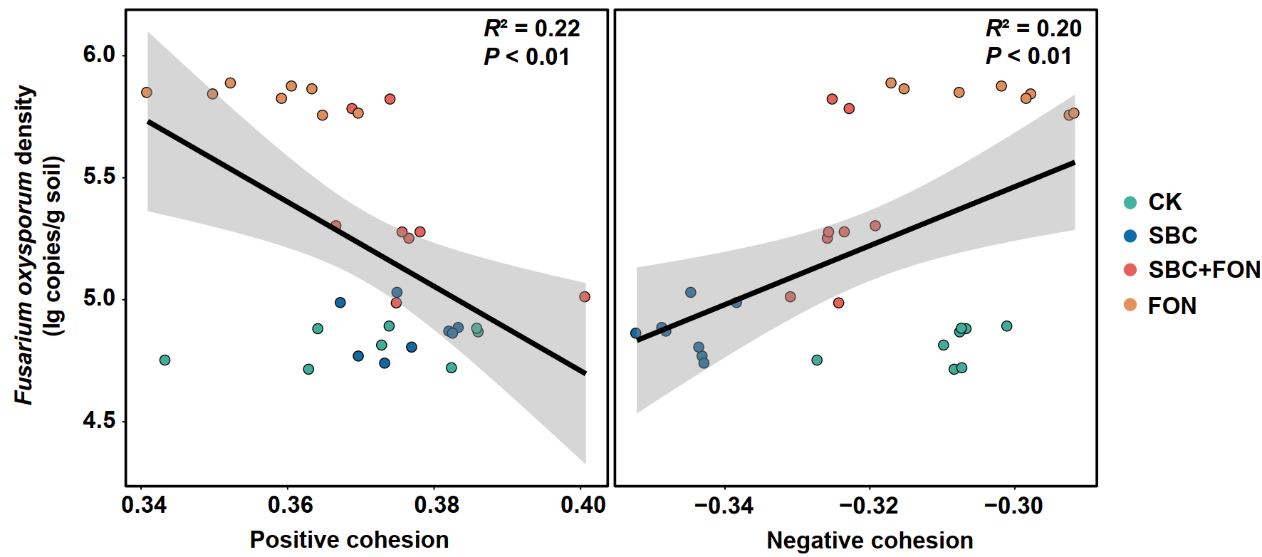
 **Fig. S10 Correlation of positive and negative network cohesion with pathogen density across inoculation treatments.** CK: ungrafted watermelon plants inoculated with sterile water; SBC: ungrafted watermelon plants inoculated with SynCom; SBC+FON: ungrafted watermelon plants inoculated with SynCom and *F. oxysporum*; FON: ungrafted watermelon plants inoculated with *F. oxysporum*.


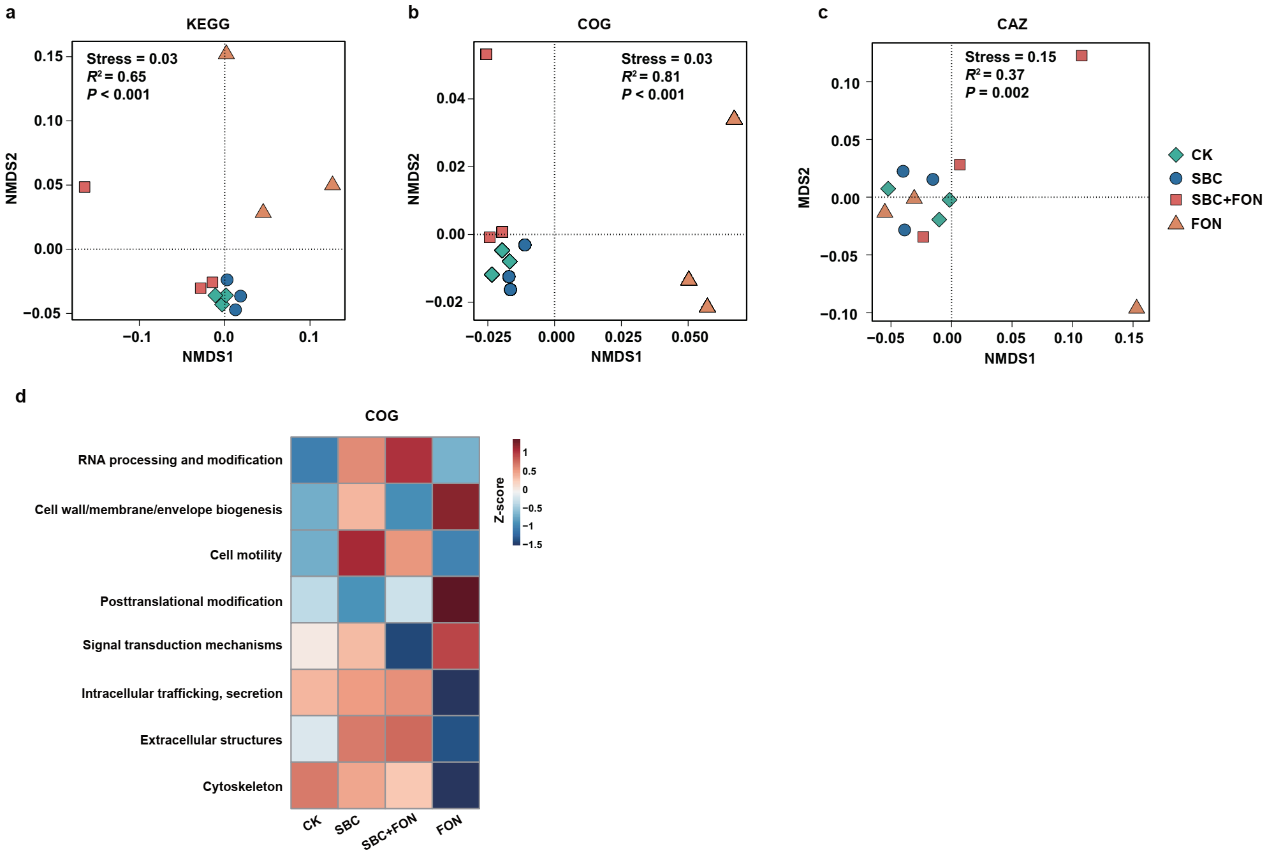
 **Fig. S11 Differential abundance analysis of KEGG, carbohydrate-active enzymes (CAZ) and clusters of orthologous groups of proteins (COG).** (a-c) NMDS analysis of KEGG, COG and CAZ. (d) Abundance of COG genes in each treatment. CK: ungrafted watermelon plants inoculated with sterile water; SBC: ungrafted watermelon plants inoculated with SynCom; SBC+FON: ungrafted watermelon plants inoculated with SynCom and *F. oxysporum*; FON: ungrafted watermelon plants inoculated with *F. oxysporum*.


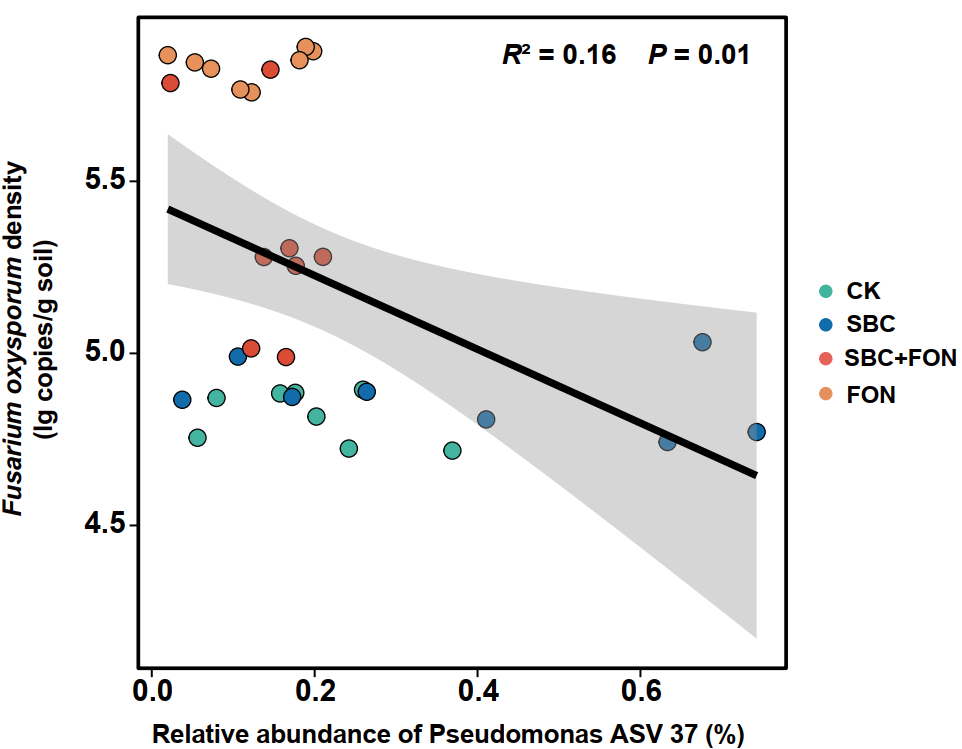
 **Fig. S12 Correlation of relative abundance of *Pseudomonas* ASV 37 with pathogen concentration.** CK: ungrafted watermelon plants inoculated with sterile water; SBC: ungrafted watermelon plants inoculated with SynCom; SBC+FON: ungrafted watermelon plants inoculated with SynCom and *F. oxysporum*; FON: ungrafted watermelon plants inoculated with *F. oxysporum*.


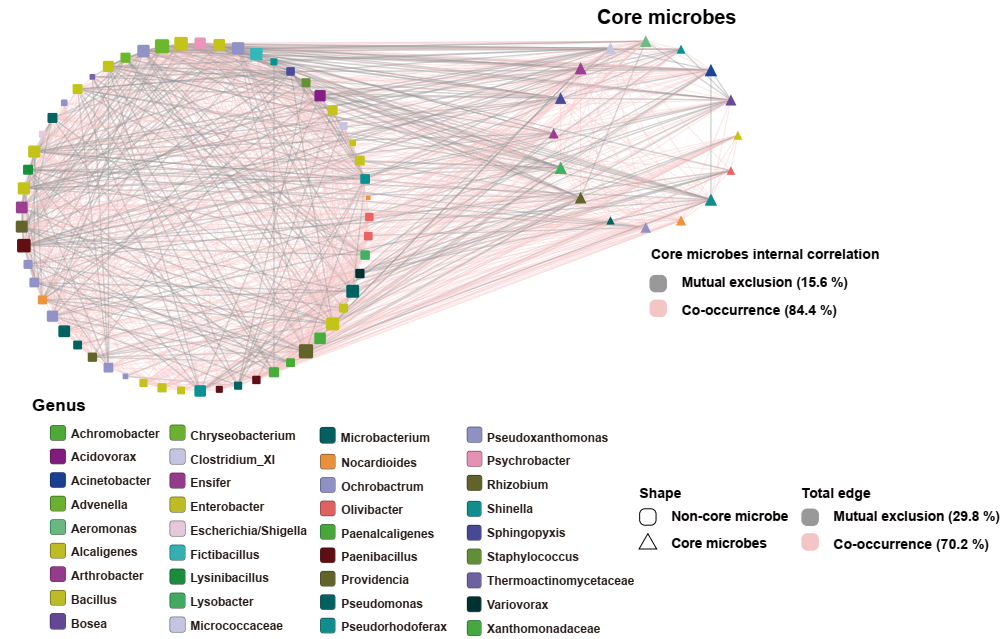
 **Fig. S13 Network of co-occurring ASVs based on correlation analysis in soils with different fertility gradients.** Core microbes exhibit strong positive correlations among them. The size of each node is proportional to the relative abundance of the ASV. Red edges represent co-occurrence between two ASVs, and gray edges represent mutually excluded ASVs. Nodes are colored according to genus taxonomy, with core microbe nodes indicated as triangles.


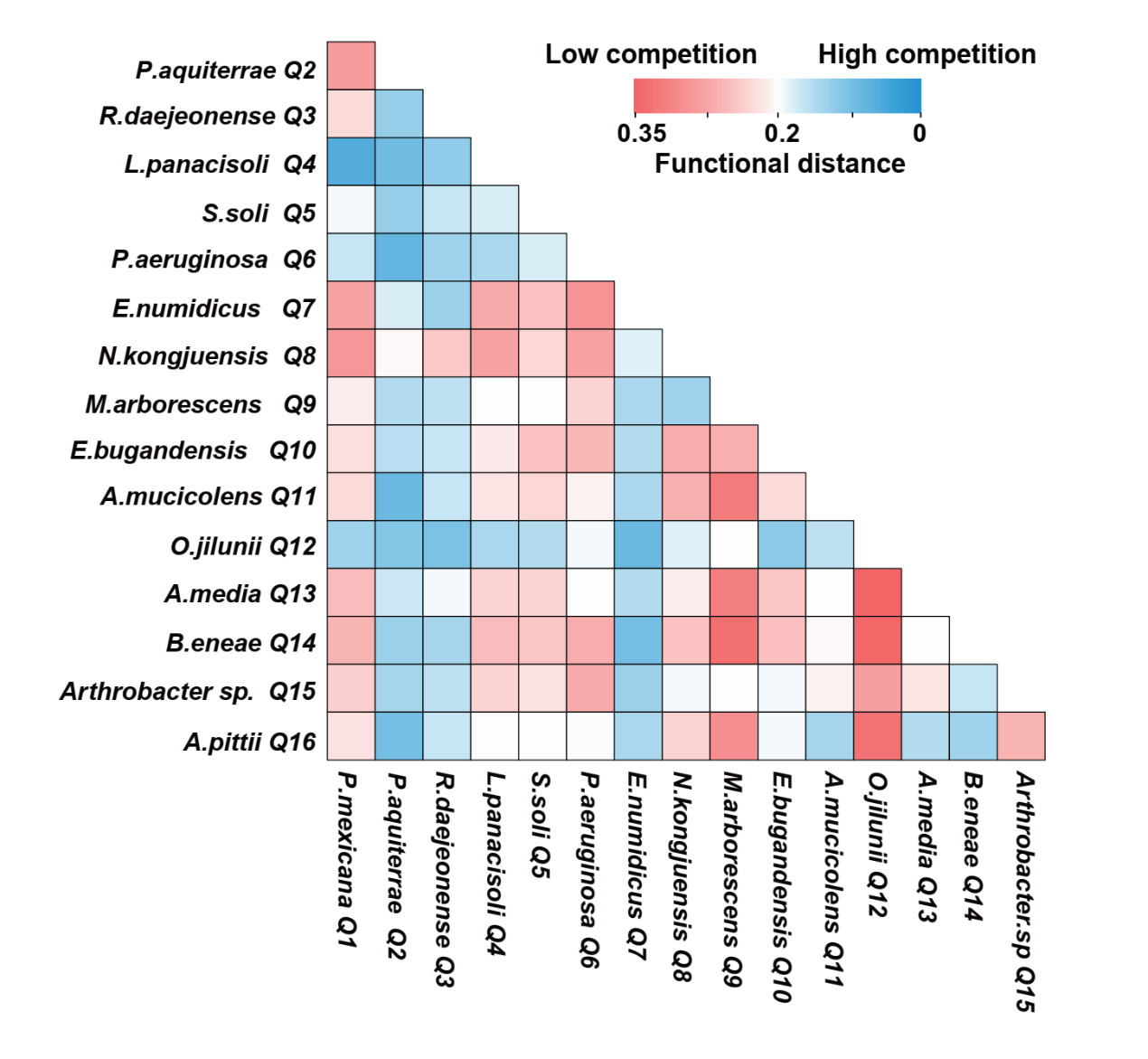
 **Fig. S14** **Functional distance between different strains.** A larger functional distance indicates a smaller overlap of metabolic networks and reduced competition between the two strains. For detailed calculation methods, see Supplementary Methods 1.12.

**
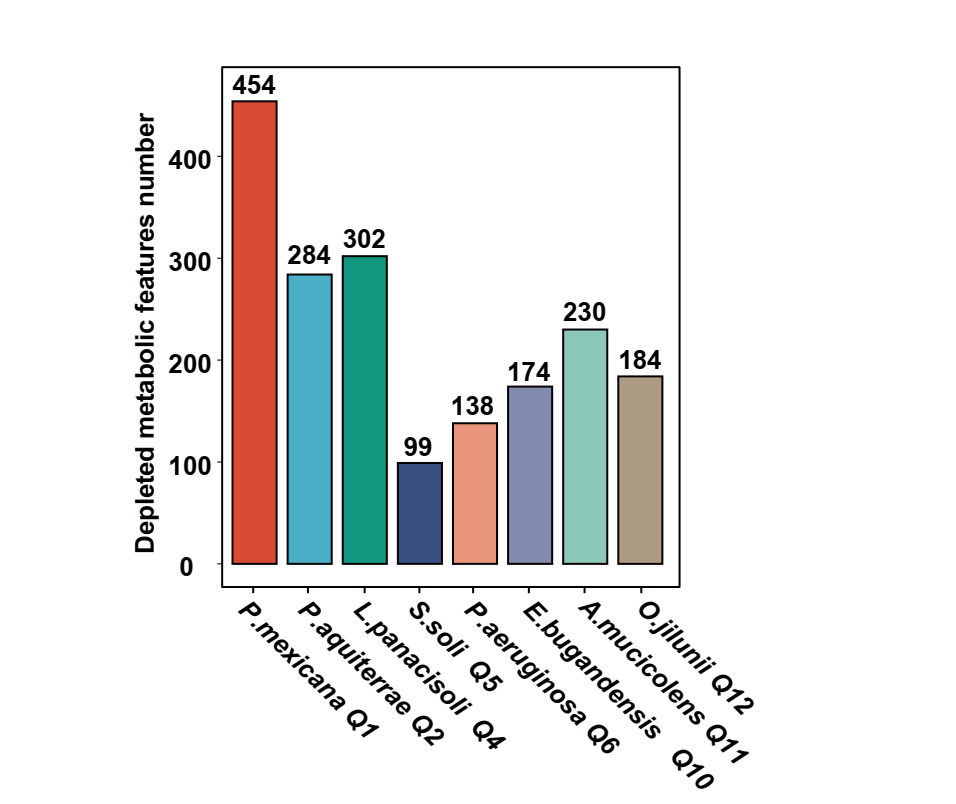
Fig. S15 Bar plot shows the total number of metabolic features significantly depleted (compared to the fresh medium; *P* < 0.05) in the fresh NB medium for each bacterial strain.**
